# The effect of exercise training on quality of life in people with chronic kidney disease requiring dialysis. A systematic review with meta-analysis

**DOI:** 10.1007/s40620-025-02245-1

**Published:** 2025-03-28

**Authors:** Annette Traise, Gudrun Dieberg, Elizabeth Degotardi, Bailey Hart, Fiza Kaippilly, Darcy McInnes, Melissa J. Pearson, David Ryan, Neil A. Smart

**Affiliations:** Clinical Exercise Physiology, School of Science and Technology, University of New England, Armidale, NSW 2351 Australia

**Keywords:** Chronic kidney disease, Dialysis, Exercise, Quality of life

## Abstract

**Background:**

Chronic kidney disease (CKD) is a debilitating condition associated with poor health outcomes, including reduced quality of life (QoL), frequent hospitalisation and premature mortality.

**Aim:**

This study aimed to determine the effect of exercise training on health-related QoL in individuals with CKD requiring dialysis, focusing on mental health scores. Secondary aims included analysing the effect of exercise modality, intensity, and delivery context to maximise exercise training benefits for QoL. Additionally, differences in mental component summary and physical component summary scores using CKD-specific generic QoL patient reported outcome measures were examined.

**Methods:**

A systematic search of MEDLINE, EMBASE, the Cochrane Library of Controlled Trials, CINAHL, and SPORTDiscus up to November 14th, 2024, identified randomised controlled trials (RCTs) comparing exercise training to usual care in CKD patients requiring dialysis. Twenty-five RCTs met the inclusion criteria and were pooled for meta-analyses.

**Results:**

Pooled analysis revealed significant improvements in QoL scores for mental component summary (MD 3.33 [1.24, 5.41], *p* = 0.002) and physical component summary (MD 3.75, [2.28, 5.23], *p* < 0.00001) compared to the usual care. A statistically significant improvement in the mental component summary was found for aerobic training (*p* = 0.02) and resistance training (*p* = 0.04). Moderate intensity (*p* = 0.003), an intervention duration of 12–26 weeks (*p* = 0.0004), interdialytic delivery (*p* = 0.003), intradialytic delivery (*p* = 0.03) and supervised training (*p* = 0.002) all demonstrated statistically significant improvements in mental component summary. The short form (SF)-36 demonstrated significant improvements in mental component summary (MD 4.15 [1.54, 6.76], *p* = 0.002), while the kidney disease QoL patient-reported outcome measure did not show significant improvement (*p* = 0.33).

**Conclusions:**

Supervised, inter-dialytic or intra-dialytic exercise, including aerobic or resistance training at a moderate intensity for up to 26 weeks, can significantly improve mental component summary scores in individuals with stage 5 CKD on dialysis.

**Graphical Abstract:**

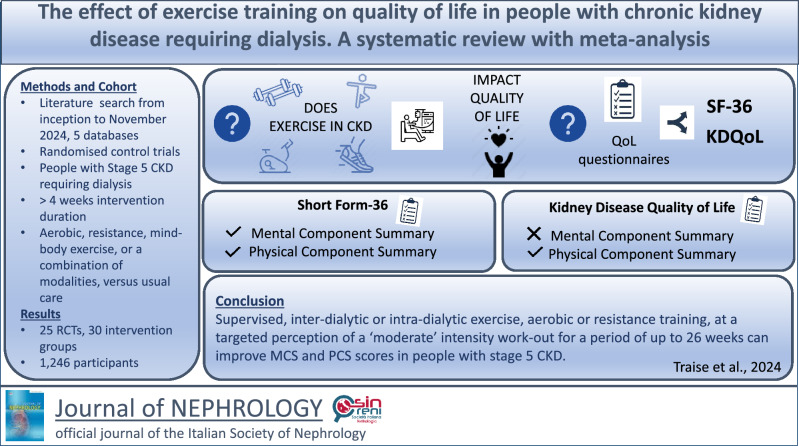

**Supplementary Information:**

The online version contains supplementary material available at 10.1007/s40620-025-02245-1.

## Introduction

Chronic kidney disease (CKD) is a debilitating illness characterised by poor health, loss of independent living, frequent hospitalisation, multiple organ failure, poor quality of life (QoL), and poor survival [1, 2]. Many people with CKD, especially those requiring dialysis, suffer from one or more comorbidities, all of which generally lead to poor health outcomes, further impacting QoL [3, 4]. Lifestyle modifications—cessation of smoking, a healthy diet, reduction of alcohol intake and being more physically active (SNAP—Smoking, Nutrition, Alcohol and Physical Activity), along with stress and sleep management, can have a positive effect on CKD health outcomes [5]. Previous studies and reviews on exercise training have shown improvements in fitness capacity, muscle strength, appetite, and inflammation, which in turn, have been associated with functional independence, reduced morbidity, and improved health-related quality of life (HRQoL) [6–9].

Quality of life refers to a person’s perception of their own physical, mental, social functioning and well-being, such as being able to care for themselves (dressing, bathing), their ability to work (whether paid or not) and to interact socially [10]. HRQoL applies specifically to the effects of disease, injury or treatment as reported by the individual, and can also include perceptions from families and caregivers [11]. There are numerous factors stemming from living with CKD that can affect a person’s HRQoL, including anaemia, pain, fatigue, sleep disturbance, diet limitations, medication side effects, depression, anxiety, and grief [12–14]. HRQoL generally declines as symptoms increase, and for those on dialysis, the hours required to dialyse several times a week, including transport, and the restrictions regular dialysis imposes on the ability to work, socialise and travel, as well as a reduction in income and increased expenses, can have a compounding effect [15].

Patient-reported outcome measures, such as questionnaires used to assess QoL, are common and useful instruments that can inform and improve patient management [16]. Several different patient reported outcome measures are used to collect and assess information on HRQoL change following a programme of exercise training. In CKD, the Short Form 36 (SF-36) has, to date, been the most commonly utilised QoL questionnaire [10, 17]. A generic, but valid and reliable patient-reported outcome measure, the SF-36, provides 10 scores; eight scores measuring aspects of perceived health and two summary scores, the mental component summary and the physical component summary, calculated from individual domain scores. Higher summary scores indicate better QoL. In CKD QoL assessment, the validated Kidney Disease Quality of Life (KDQoL)^, PROM^, which includes the SF-36 as a generic core element along with a series of kidney disease-specific questions, is also frequently used, given its disease-specific context.

It has been shown that a decrease in mental component summary and physical component summary scores is associated with an increased risk of hospitalisations and mortality, and that the restoration of these outcomes through interventions should be a priority [18]. Previous studies have summarised the effect of exercise training on mental component summary and physical component summary and have consistently shown that physical component summary scores have improved with the inclusion of exercise training; however, this trend has not been the same for mental component summary scores [19–26]. Data from 10 reviews prior to 2021, and the 2022 Bernier-Jean Cochrane Review, reveal that only 3 reported an improvement in mental component summary scores [24]. Prior reviews vary in their primary focus, some focusing on a specific modality, e.g., aerobic training [27], some on the timing of exercise—intra-dialytic [21, 25], inter-dialytic [26], or both [20]. Given the largely neutral and somewhat inconsistent findings on the benefits of exercise training for mental component summary scores and the recent publication of numerous studies, an updated exploration of data from randomised controlled trials (RCTs) comparing exercise training to a control is warranted.

The primary aim of this study was to determine the effect of exercise training on the mental component summary scores derived from the SF-36 QoL questionnaire, and its shorter versions, in individuals undergoing dialysis for CKD. Secondary aims included analysing how different delivery methods of exercise training impacted mental component and physical component summary scores. This analysis evaluated the influence of exercise modality, the intensity and duration of the exercise intervention, and the context of the exercise intervention—whether conducted during (inter-dialytic) or between (intra-dialytic) dialysis sessions and supervised or unsupervised training. The sub-analyses aimed to identify specific conditions that may have the potential to maximise the benefits of exercise to improve HRQoL for individuals with Stage 5 CKD on dialysis. Our final aim was to examine the mental component summary and physical component summary results from two frequently used patient reported outcome measures in CKD, the standalone SF-36 and the KDQoL, to consider if the additional questions in the KDQoL may have the potential to influence scores.

## Methods

A protocol for the review was registered with PROSPERO (CRD42023405446).

## Search strategy

We conducted systematic searches of MEDLINE, EMBASE, the Cochrane Library of Controlled Trials, CINAHL, and SPORTDiscus up to November 14th, 2024. The search criteria encompassed a combination of medical subject headings (MeSH), and free text terms related to CKD, kidney or renal disease, exercise or physical activity, fitness, and randomised controlled trials or RCT.

The search strategy was developed following the PICO framework [28], utilising the elements: P (Population)—individuals with CKD stage 5 requiring dialysis, allocated to an exercise training intervention group within RCTs; I (Intervention)—exercise training programmes spanning a minimum duration of > 4 weeks; C (Comparator)—individuals with CKD stage 5 requiring dialysis, allocated to a sedentary control group in RCTs focusing on exercise training intervention; and O (Outcomes)—HRQoL. The search was limited to peer-reviewed, published RCTs. A detailed description of the search strategy is provided in the Online Resources, Supplemental Table 1. Six reviewers (LD, BH, FK, DM, DR, AT) conducted the search. The results of the search were then divided into three groups with two reviewers assigned to assess article eligibility within each group. In the event of disagreement, a third reviewer was consulted.

## Study selection

The following criteria were applied for study identification and selection (1) randomised controlled (or prospective) clinical trials in CKD stage 5 requiring dialysis; (2) human studies; (3) studies must have included a usual care or no exercise group (sham exercise or passive stretching permitted); (4) the exercise intervention period was greater than four weeks. Studies of non-dialysis patients or those including participants with no diagnosis of CKD or < 18 years of age were excluded.

## Intervention

Exercise was characterised as any structured form of exercise training, including aerobic exercise, resistance training, combined training (aerobic + resistance), mind–body practices such as Tai Chi, and inspiratory muscle training.

## Outcomes

Studies qualified for inclusion in the review provided they presented data on the outcomes of mental component summary and/or physical component summary using a comparable version of the HRQoL Short Form (SF) questionnaire [29]. These versions included the SF-12, SF-36, KDQoL-36, KDQoL-SF and KDQoL-long form (LF) questionnaires. Details of commonalities and relationship of questionnaires are provided in Supplemental Fig. 1. in the Online Resources.

## Data extraction

Six reviewers (LD, BH, FK, DM, DR, AT) conducted data extraction. For each study, the following information was collected: author; year of publication; study characteristics (country, sample size, type of dialysis); participant characteristics (age, sex); intervention characteristics (modality and delivery, intensity, duration, frequency, supervision); patient reported outcome measure used; and outcomes. A standardised data extraction form was employed for this purpose. In cases where additional data or clarification was necessary, we reached out to the study authors.

## Data synthesis

Statistical analyses were performed using the RevMan V5.4 software [30]. Individual meta-analyses were completed for continuous data by using the mean baseline follow-up change and standard deviation (SD). If the mean change was not reported, we calculated it by subtracting the baseline mean from the mean at the intervention’s conclusion. In instances where change SDs were not provided, but exact p values or 95% confidence intervals (CIs) for within-group changes were provided, these were input into RevMan to compute the change SDs. In the absence of exact p values or 95% CIs, SDs were estimated using the Cochrane formula for standard deviation: SD = square root [(SD_pre-treatment_)^2^ + (SD_post-treatment_) ^2^—(2rSD_pre-treatment_ x SD_post-treatment_) [31], with a correlation coefficient of 0.5, which is considered a conservative value. When the standard error of the mean (SEM) was reported instead of the SD, it was converted to SD [32]. Additionally, data presented as median and interquartile range were converted to mean and SD following the method described by Wan [33].

The analysis utilised a random effects inverse variance model, with the mean difference (MD) serving as the measure of effect. This approach accommodates the variability and the potential influence of unrecorded factors, such as the participants’ diverse ages, health statuses and the varying intensity of the interventions across the included studies [34]. The random effects model makes less stringent assumptions about the consistency of effects and imposes fewer restrictions compared to a fixed effect model, making it a more suitable choice for meta-analyses, producing a more realistic estimate of the uncertainty in the overall treatment effect [34, 35].

We considered statistical significance at the 5% level and reported pooled mean results with 95% CIs. In studies with multiple intervention groups and a control group, each intervention group was considered separately, and the control group’s sample size was divided by the number of intervention groups to prevent sample size inflation. When multiple time points during the intervention were reported, we only extracted data comparing the baseline and the end of the intervention. This review uses a similar method to [36] and the methods description partly reproduces their wording.

## Sub-analyses

Sub-analyses of the mental component summary and physical component summary were conducted for exercise modality, intensity, intervention duration, timing of intervention, and the level of supervision. The type of patient-reported outcome measure utilised was also examined.

Exercise modalities analysed included any form of aerobic training (e.g. walking, cycling), resistance training (e.g. TheraBand, hand weights), combined aerobic and resistance training and inspiratory muscle training. We categorised our exercise training intensity data into three levels based on the BORG rating of perceived exertion scale [37] (see Online Resources Supplemental Table 2). For those studies that did not report the total time per exercise session, or reported intensity using methods other than the BORG rating of perceived exertion, such as the OMNI exertion scale, percentage of repetition maximum or maximal exercise capacity, peak tolerance, or fatigue level, we graded these using equivalent estimations [38–40].

Intervention durations analysed included four to 12 weeks, 12 weeks to six months, and greater than six months. We analysed the timing of intervention relative to the timing of dialysis. We categorised the intervention to be intra-dialytic when exercise was undertaken either immediately before, during, or immediately after a dialysis session, or inter-dialytic, which included exercise completed at times separate from dialysis sessions. Levels of intervention supervision analysed included supervised and unsupervised.

We also completed a sub-analysis of mental component summary and physical component summary based on the patient-reported outcome measure utilised, examining the HRQoL with the SF-36 as a standalone set of questions and KDQoL which incorporates the SF-36 or SF-12, and a further set of questions related to kidney disease. This sub-analysis aimed to investigate whether the additional questions in the KDQoL may have influenced the mental component summary and physical component summary scores.

## Sensitivity analysis

Sensitivity analysis using a leave-one-out method was conducted to identify studies that exert a larger than normal impact on results. We also analysed the outcomes by removing the low-quality studies as assessed by the TESTEX (‘Tool for the assEssment of Study qualiTy and reporting in EXercise’) tool, which is tailored for assessing exercise training in trials involving individuals with chronic diseases [41], to determine if these studies introduced bias or affected the accuracy of our results.

## Heterogeneity and publication bias

The heterogeneity of the included studies was assessed using RevMan V5.4 software. To determine the level of agreement among the studies, the *I*^2^ test was utilised. An *I*^2^ value below 25% signals a low risk of heterogeneity, whereas an *I*^2^ value above 75% denotes a high likelihood. *I*^2^ values that lie between 25 and 75% reflect a moderate likelihood of heterogeneity [32]. Additionally, these *I*^2^ statistics, in conjunction with an examination of Egger funnel plots, were used to assess the overall heterogeneity and to gauge the potential for publication bias [42].

## Study quality

The evaluation of the quality of the included studies was performed using two evaluation tools. The Cochrane Risk of Bias (RoB2) tool was applied to categorise the studies as having ‘low risk of bias’, ‘some concerns’, or ‘high risk’ of bias [43]. Studies identified as ‘high risk’ were deemed to be of lower quality. The assessment was conducted independently by four reviewers (ED, BH, DM and AT). The same reviewers utilised TESTEX. The TESTEX tool uses a 15-point scale, dividing points between study quality (up to 5 points) and reporting (up to 10 points). Studies scoring less than 10 points on this scale were considered to be of lower quality [41].

## Results

We retrieved 858 published articles using the defined search criteria and nominated databases. Four additional studies were identified from reference lists. After removing duplicates and excluding articles based on title and/or abstract, the remaining articles were reviewed based on eligibility criteria, of which 25 were included for analysis. The Preferred Reporting Items for Systematic Reviews and Meta-Analyses (PRISMA) flow diagram (Online Resources, Supplemental Fig. 2) details the selection process. Details of excluded studies are supplied in Online Resources, Supplemental Table 3.

## Characteristics

Of 25 RCTs included in our review 22 involved a single exercise intervention group and a control group. Two studies included three types of exercise compared to a control group [44, 45], and one study included both an inter-dialytic and an intra-dialytic intervention group compared to a control group [46], resulting in 30 intervention groups. Twenty-four studies (29 intervention groups) reported mental component summary scores, with 25 studies (30 intervention groups) providing physical component summary scores. There were 1246 participants in total, (656 assigned to exercise groups and 590 to control groups). The mean age of the participants was 57.5 years (ranging from 40.7 to 70.3 years), with 65.4% of participants being male (range of 44.5% to 100%, with one study [47] not providing this data). Brazil, Greece and Korea, each conducted three studies; two studies were each conducted in the Republic of China, the United States of America, the United Kingdom and Japan; and one study conducted in each of Australia, Canada, the Czech Republic, Denmark, France, Iran, Thailand and Tunisia. Table 1 provides detailed characteristics on included studies.

**Table 1:** Summary of included studies

Author, date, country, trial	Date of trial	Study population	No analysed Ex/C	% male	Mean age (years)	Training modality, frequency, duration, intensity, supervision	Data collection (weeks)	Drop out Ex/C	Data extracted
Chen J, 2010 [55] United States of America	2005—2007	HD	22/22	52.2	69	RT, 2/week, 24 weeks, moderate intensity, supervised	0, 24	3/3	HRQOL SF-36
Dobsak, 2012 [56] Czech Republic	N/S	HD	11/5	50	58.8	AT, 3/week, 20 weeks, moderate intensity, supervised	0, 20	0/0	HRQOL SF-36
Frih, 2017 [69] Tunisia	2012—2013	HD	21/20	100	64.7	CT, 4/week, 16 weeks, moderate intensity	0, 16	7/2	HRQOL SF-36
Giannaki, 2013 [66] Greece	2007—2009	HD	15/7	72.7	56.5	AT, 3/week, 26 weeks, moderate intensity, supervised	0, 26	1/1	HRQOL SF-36
Graham-Brown, 2021 [63] United Kingdom CYCLE-HD	2014 -2018	HD	65/65	73.1	57.2	AT, 3/week, 26 weeks, moderate intensity, supervised	0, 26	14/15	HRQOL SF-12
Greenwood, 2021 [64] United Kingdom PEDAL	2019—2020	HD	116/127	44.5	60.1	CT: AT 3/week, RT 2/week, 26 weeks, light intensity, supervised	0, 26	59/33	KDQOL-36
Hristea, 2016 [47] France	N/S	HD	7/9	NS	69.8	AT, 3/week, 26 weeks, light intensity, supervised	0, 26	3/2	HRQOL SF-36
Huang, 2020 [51] Republic of China	2019	HD	16/16	71.8	40.7	CT, 3/week, 24 weeks, moderate intensity, supervised	0, 24	6/6	KDQOL-SF
Jamshidpour 2020 [62] Iran	N/S	HD	15/13	71.4	61.9	CT, 3/week, 8 weeks, vigorous intensity, supervised	0, 8	0/2	HRQOL SF-36
Jeong, 2019 [49] USA IHOPE	2011—2016	HD	29/34	58	54.8	AT, 3/week, 52 weeks, moderate intensity	0, 52	20/10	HRQOL SF-12
Kim, 2022 [53] Korea	N/S	HD	21/21	51.3	57.1	AT, 3/week, 12 weeks, vigorous intensity, supervised	0, 12	3/0	KDQOL-SF
Koh, 2010 [46] Australia	2006—2007	HD	15/8	60.8	51.9	AT, 3/week, 26 weeks, moderate intensity, unsupervised (centre-based)	0, 26	6/3	HRQOL SF-36
	2006—2007	HD	15/8	65.2	51.8	AT, 3/week, 26 weeks, moderate intensity, supervised (home-based)	0, 26	5/3	HRQOL SF-36
Lee, 2020 [45] Korea	N/S	HD	11/4	46.6	55.1	AT, 3/week, 12 weeks, moderate intensity, supervised	0, 12		KDQOL-SF
	N/S	HD	10/4	57.1	53.5	RT, 3/week, 12 weeks, moderate intensity, supervised	0, 12		KDQOL-SF
	N/S	HD	12/5	58.8	58	CT, 3/week, 12 weeks, moderate intensity, supervised	0, 12		KDQOL-SF
Matsufuji, 2015 [58] Japan CHAIR	N/S	HD	6/11	65.5	68.7	AT, 3/week, 12 weeks, mild to moderate intensity, supervised	0, 12	5/1	HRQOL SF-36
Maynard, 2019 [50] Brazil	N/S	HD	20/20	55	45.5	CT, 3/week, 12 weeks, moderate intensity, supervised	0, 12	2/3	KDQOL-SF
Molsted, 2004 [48] Denmark	N/S	HD	11/9	66.7	55.3	CT, 3/week, 12 weeks, vigorous intensity, supervised	0, 12	11/2	HRQOL SF-36
Ouzouni, 2009 [67] Greece	N/S	HD	19/14	77.1	48.8	CT, 3/week, 43 weeks, moderate intensity, supervised	0, 43	1/1	HRQOL SF-36
Pereira, 2022 [54] Brazil	2019–2020	HD	40/40	62.5	70.3	AT, 3/week, 12 weeks, moderate intensity, supervised	0, 12	9/9	KDQOL-SF
Rosa, 2018 [59] Brazil	2013–2014	HD	28/24	67.3	55.7	RT, 3/week, 12 weeks, vigorous intensity, supervised	0, 12	6/5	KDQOL-SF
Samara, 2016 [68] Greece	N/S	HD	15/12	88.8	48.2	AT, 3/week, 16 weeks, moderate intensity, supervised	0, 16	2	HRQOL SF-36
Song, 2012 [57] Korea	2010	HD	20/20	50	53.5	RT, 3/week, 12 weeks, moderate intensity, supervised	O, 12	2/2	HRQOL SF-36
Thompson, 2016 [44] Canada	N/S	HD	8/3	90	62.1	AT, 3/week, 12 weeks, moderate intensity, supervised	0, 12	1/2	KDQOL-36
	N/S	HD	7/2	89	57.4	RT, 3/week, 12 weeks, moderate intensity, supervised	0, 12	1/1	KDQOL-36
	N/S	HD	8/3	55	57.3	CT, 3/week, 12 weeks, moderate intensity, supervised	0, 12	1/1	KDQOL-36
Uchiyama, 2019 [61] Japan	2016–2018	PD	24/23	74.5	64.1	CT, 3/week, 12 weeks, moderate intensity, unsupervised	0, 12	2/1	KDQOL-36
Yuenyongchaiwat, 2021 [65] Thailand	N/S	HD	23/21	59.1	52.4	IMT, 3/week, 8 weeks, moderate intensity, supervised	0, 8	2/4	KDQOL-36
Zhang, 2020 [52] Republic of China	2018	HD	43/44	60.9	58.32	RT, 3/week, 12 weeks, moderate intensity, supervised	0, 12	2/1	KDQOL-SF

ACTINUT trial

*CHAIR trial* change haemodialysis patients’ activity and impaired functions by chair stand exercise

*CYCLE-HD trial* improving cardiovascular outcomes in haemodialysis patients using a structured programme of exercise

*IHOPE trial* the intra-hemodialytic oral protein and exercise

*PEDAL trial*: PrEscription of intra-dialytic exercise to improve quality of Life

*HRQOL SF*-12 health related quality of life short form 12 health questionnaire, *HRQOL SF*-36 health related quality of life short form 36 health questionnaire, *IMT* inspiratory muscle training, *KDQOL*-*SF* kidney disease quality of life short form health questionnaire, *KDQOL*-36 kidney disease quality of life short form 36 health questionnaire, *N*/*S* not stated, *PD* peritoneal dialysis

The primary reported causes of CKD were hypertensive nephropathy, diabetic nephropathy, glomerulonephritis and polycystic kidney disease [44, 46, 48–54], and co-morbidities were diabetes, hypertension, coronary artery, cardiovascular and ischaemic heart disease, stroke, and hyperlipidaemia [44, 54–64]. The majority of the studies were completed on patients undergoing haemodialysis, with one conducted on people on peritoneal dialysis [61]. Five studies had the control group undergoing sham exercises or simple stretches [44, 55, 58, 59, 65]. One study focused on malnourished older patients [47], with both groups able to receive either oral nutritional supplements or intra-dialytic parenteral nutrition, and another included a nutritional supplement for the exercise intervention group only [49]. All participants in one study were type two diabetics [62], in another study only participants experiencing restless legs syndrome were included [66], and one study enrolled participants aged over 60 years only [58]. Of the 25 studies, 17 utilised the HRQoL SF-36, or shortened version questionnaire (two used the SF-12 [49, 63], 15 used the SF-36 [44, 46–48, 53, 55–59, 62, 66–69]) and eight utilised the KDQoL (five used the KDQoL-SF [45, 50, 52, 54, 64] and three the KDQoL-36 [51, 61, 65]).

## Intervention details

For comprehensive detailed intervention characteristics, refer to Online Resources Supplemental Table 4. Among the 30 intervention groups, aerobic training was employed in 13, combined training in 10, resistance training in six, and inspiratory muscle training in one. We assigned an exercise intensity of ‘light’ to two intervention groups, ‘moderate’ to 24, and ‘vigorous’ to four.

Thirteen studies encompassed an intervention duration of 4 to 12 weeks, ten studies were between 12 and 26 weeks, and two were between 26 and 52 weeks. Exercise session frequency was reported as three times a week for 24 of the studies, with one reporting a frequency of three to four times per week. Exercise session durations ranged from 15 to 90 min. Seven intervention groups underwent inter-dialytic exercise and 23 intra-dialytic. The exercise sessions were supervised in 28 of the intervention groups, and unsupervised in two (Online Resources Supplemental Table 4).

## Outcomes

A detailed summary of the meta-analyses and sub-analyses is provided in Table 2.

**Table 2:** Summary of meta-analyses and sub-analyses of Quality of Life Short-form 36 Physical Component Summary and Mental Component Summary

Category	Number of studies (intervention groups)	Participants exercise/control	Result: MD (95% CI), *p*, *I*^2^ MCID
Meta-analyses			
Mental component summary	24 (29)	542/470	MD 3.33 [1.24, 5.41], *p* = 0.002, *I*^2^ = 48%*
Physical component summary	25 (30)	656/590	MD 3.75 [2.28, 5.23], *p* < 0.00001, *I*^2^ = 22%*
Sub-analyses by exercise modality			
Aerobic training			
Mental component summary	12 (13)	244/212	MD 4.57 [0.65, 8.49], *p* = 0.02, *I*^2^ = 64%*
Physical component summary	12 (13)	244/212	MD 4.43 [1.02, 7.83], *p* = 0.01, *I*^2^ = 45%*
Resistance training			
Mental Component Summary	6 (6)	130/116	MD 3.07 [0.09, 6.04], *p* = 0.04, *I*^2^ = 0%*
Physical component summary	6 (6)	130/116	MD 4.73 [0.57, 8.89], *p* = 0.03, *I*^2^ = 34%*
Combined training			
Mental component summary	9 (9)	145/121	MD 2.98 [− 0.53, 6.49], *p* = 0.10, *I*^2^ = 47%
Physical component summary	10 (10)	259/241	MD 3.78 [2.04, 5.51], *p* < 0.0001, *I*^2^ = 0%*
Inspiratory muscle training			
Mental component summary	1 (1)	23/21	MD -0.81 [− 21.62, 20.00], *p* = 0.94, *I*^2^ = NA
Physical component summary	1 (1)	23/21	MD 0.73 [-3.36, 4.82], *p* = 0.73, I^2^ = NA
Sub-analyses by intensity of exercise—Borg Rating of Perceived Exertion Scale			
Light			
Mental component summary	1 (1)	7/9	MD 39.76 [18.72, 60.80], *p* = 0.0002, *I*^2^ = NA*
Physical component summary	2 (2)	121/129	MD 14.51 [− 12.60, 41.63], *p* = 0.29, *I*^2^ = 89%
Moderate			
Mental component summary	19 (24)	461/396	MD 3.18 [1.09, 5.28], *p* = 0.003, *I*^2^ = 45%*
Physical component summary	19 (24)	461/396	MD 3.42 [1.98, 4.86], *p* < 0.00001, *I*^2^ = 7%*
Vigorous			
Mental component summary	4 (4)	74/65	MD 2.25 [− 2.07, 6.58], *p* = 0.31, *I*^2^ = 0%
Physical component summary	4 (4)	74/65	MD 6.06 [2.09, 10.03], *p* = 0.003, *I*^2^ = 0%*
Sub-analyses by duration of exercise intervention			
> 4 to 12 weeks			
Mental component summary	13 (17)	306/265	MD 1.13 [− 0.89, 3.15], *p* = 0.27, *I*^2^ = 0%
Physical component summary	13 (17)	306/265	MD 2.72 [0.98, 4.46], *p* = 0.002, *I*^2^ = 0%*
> 12 to 26 weeks			
Mental component summary	9 (10)	188/153	MD 7.25 [3.25, 11.25], *p* = 0.0004, *I*^2^ = 68%*
Physical component summary	10 (11)	302/273	MD 5.84 [3.04, 8.63], *p* < 0.0001, *I*^2^ = 37%*
> 26 weeks			
Mental component summary	2 (2)	48/52	MD 2.07 [− 1.56, 5.70], *p* = 0.26, *I*^2^ = 0%
Physical component summary	2 (2)	48/52	MD 1.58 [-3.65, 6.80], *p* = 0.55, *I*^2^ = 67%
Sub-analyses by schedule of exercise intervention			
Interdialytic			
Mental component summary	7 (7)	111/101	MD 5.78 [1.99, 9.56], *p* = 0.003, *I*^2^ = 38%*
Physical component summary	7 (7)	111/101	MD 6.80 [4.15, 9.44], *p* < 0.00001, *I*^2^ = 0%*
Intradialytic			
Mental component summary	18 (22)	431/369	MD 2.50 [0.21, 4.79], *p* = 0.03, *I*^2^ = 40%*
Physical component summary	19 (23)	545/489	MD 2.94 [1.30, 4.57], *p* = 0.0004, *I*^2^ = 22%*
Sub-analyses of KDQOL-36 by supervision of exercise intervention			
Supervised			
Mental component summary	23 (27)	503/439	MD 3.52 [1.33, 5.71], *p* = 0.002, *I*^2^ = 50%*
Physical component summary	24 (28)	617/559	MD 3.77 [2.19, 5.36], *p* < 0.00001, *I*^2^ = 28%*
Unsupervised			
Mental component summary	2 (2)	39/31	MD 0.19 [− 4.90, 5.29], *p* = 0.94, *I*^2^ = 0%
Physical component summary	2 (2)	39/31	MD 4.15 [− 2.05, 10.36], *p* = 0.19, *I*^2^ = 0%
Sub-analyses by patient-reported outcome measure			
Health-related quality of life short form questionnaire			
Mental component summary	17 (20)	343/293	MD 4.15 [1.54, 6.76], *p* = 0.002, *I*^2^ = 51%*
Physical component summary	17 (20)	343/293	MD 5.17 [3.00, 7.35], *p* < 0.00001, *I*^2^ = 27%*
Kidney disease quality of life questionnaire			
Mental component summary	7 (9)	199/177	MD 1.48 [− 1.50, 4.46], *p* = 0.33, *I*^2^ = 23%
Physical component summary	8 (10)	313/297	MD 1.86 [0.23, 3.49], *p* = 0.03, *I*^2^ = 0%*

*CI* confidence interval, *I*^2^ percentage of variation across studies due to heterogeneity, *MD* mean difference, *NA* not applicable, *significant p value of 0.05 or less

### Mental component summary

**Fig. 1:**
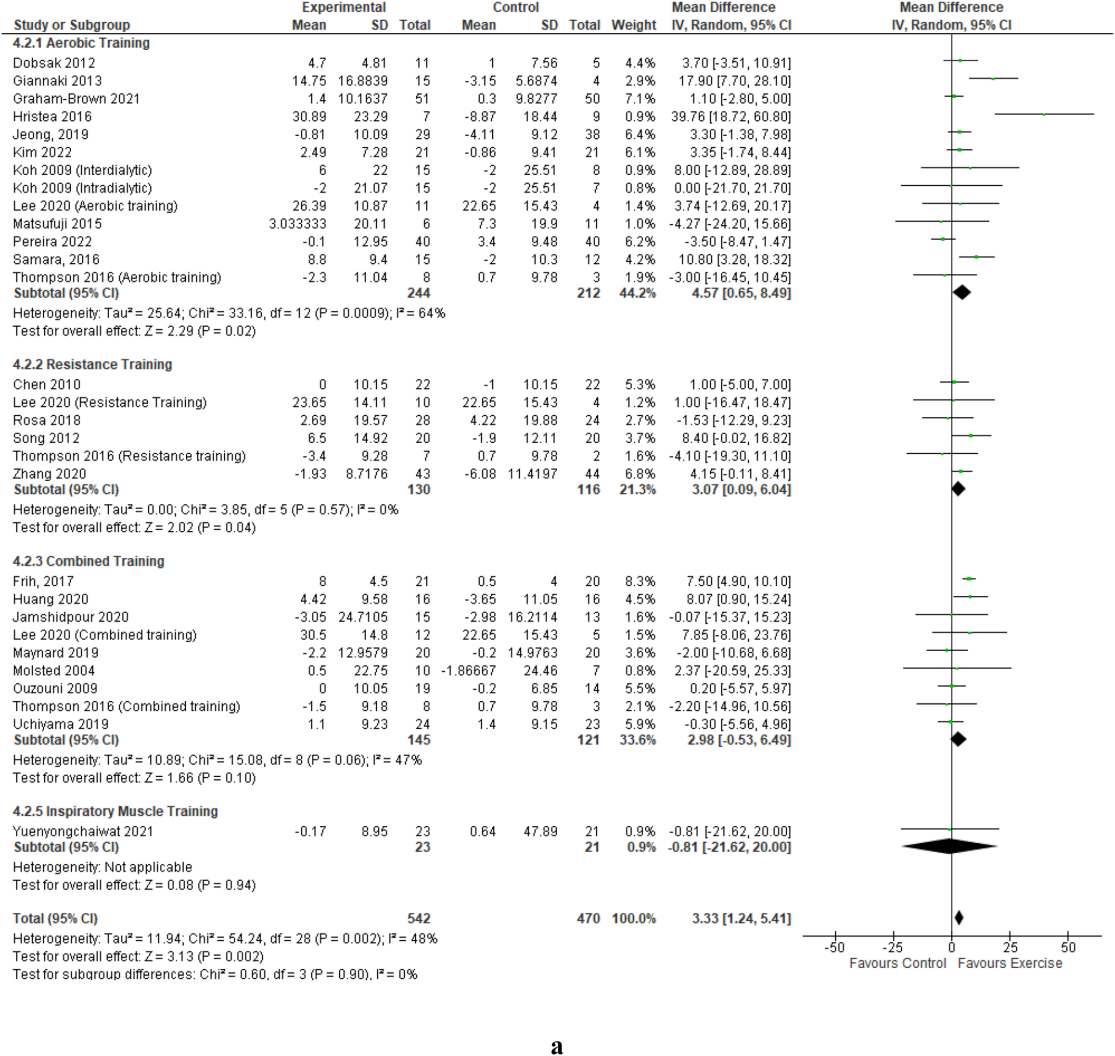

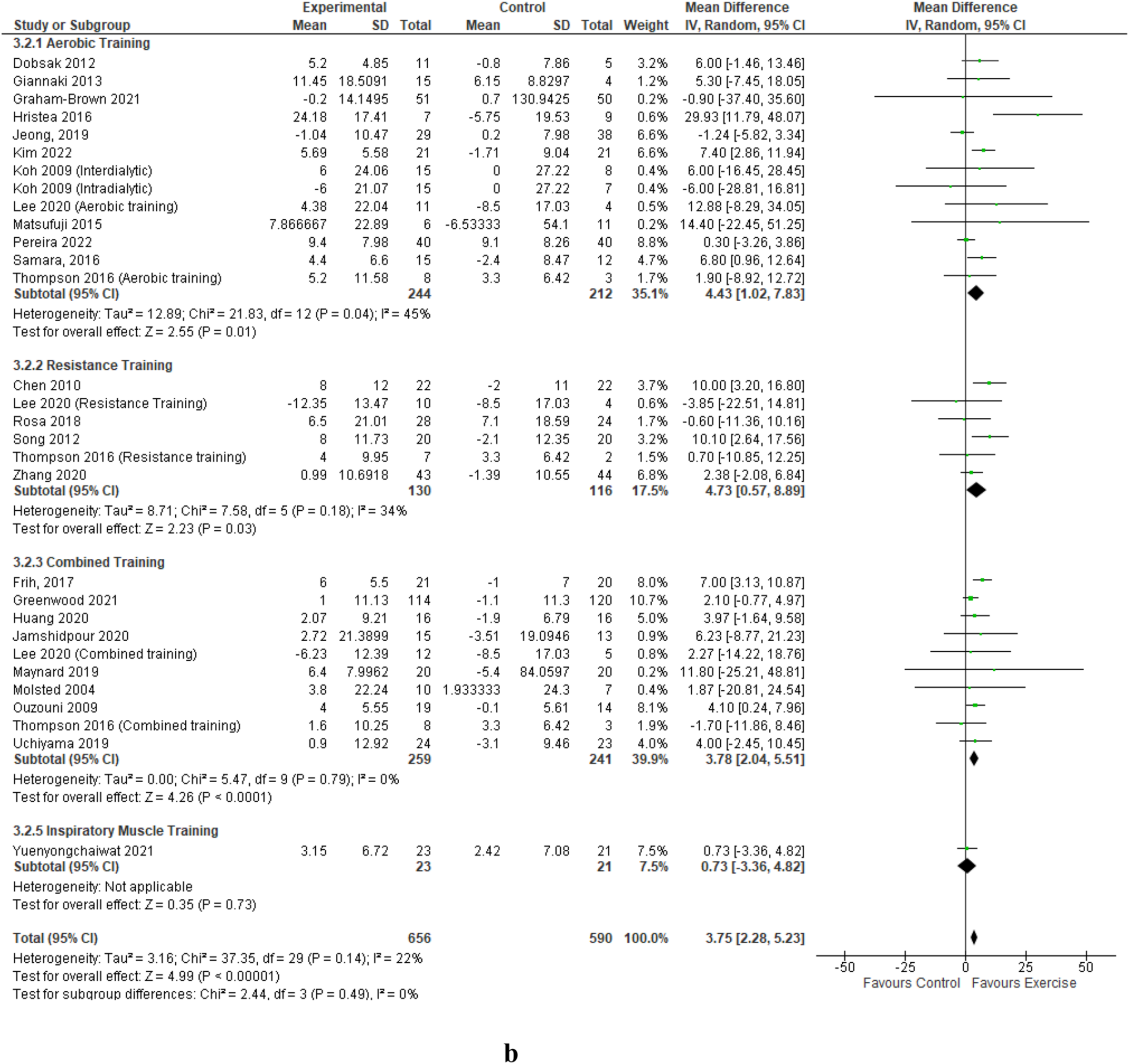
Change in Mental (**a**) and Physical (**b**) Component Summaries (score out of 100) in people with CKD stage 5 requiring dialysis with exercise as an intervention compared to a control group – by exercise modality. **a** Change in Mental Component Summary – by exercise modality. **b** Change in Physical Component Summary – by exercise modality

Data from 24 studies reporting on the mental component summary, involving 29 intervention groups and 1012 participants [44–63, 65–69] pooled for analysis revealed a statistically significant improvement in the mental component summary score in favour of the exercise training group compared to the control group (MD 3.33 (95% CI 1.24, 5.41, *p* = 0.002) (Fig. 1a). The statistically significant improvement in the mental component summary score remained when low-quality studies were removed; MD 2.93 (95% CI 0.87, 4.99, *p* = 0.005) (Online Resources Supplemental Fig. 3). Leave-one-out sensitivity analyses did not produce any statistical changes of note.

For the primary outcome of mental component summary, statistically significant improvements were found for aerobic training; MD 4.57 (95% CI 0.65, 8.49, *p* = 0.02), and resistance training; MD 3.07 (95% CI 0.09, 6.04, *p* = 0.04.) Pooled data from nine studies of combined training demonstrated a trend for improvement; MD 2.98 (95% CI − 0.53, 6.49, *p* = 0.10); however, this was not statistically significant (Fig. 1a). Pooled exercise training intensity, rated on a rating of perceived exertion scale, demonstrated statistically significant improvements for mental component summary scores for moderate intensity exercise; MD 3.18 (95% CI 1.09, 5.28, *p* = 0.003) and light intensity; however, ‘light’ only represented one study (Online Resources Supplemental Fig. 4). Mental component summary improved significantly with exercise training intervention duration of > 12 to 26 weeks; MD 7.25 (95% 3.25, 11.25, *p* = 0.0004) (Online Resources Supplemental Fig. 5).

Delivery context demonstrated statistically significant improvements in mental component summary for interdialytic exercise; MD 5.78 (95% CI 1.99, 9.56, p = 0.003) when data from seven intervention groups were pooled, and intradialytic exercise; MD 2.50 (95% CI 0.21, 4.79), *p* = 0.03) when data from 22 intervention groups were pooled (Online Resources Supplemental Fig. 6). Pooled data from 27 intervention groups demonstrated significant improvements in mental component summary from supervised training; MD 3.52 (95% CI 1.33, 5.71, *p* = 0.002), with no improvement from unsupervised training, although only two intervention groups were unsupervised (Online Resources Supplemental Fig. 7).

### Physical component summary

Data from 25 studies [44–69] reported on the physical component summary score, involving 30 intervention groups and 1246 participants. The pooled analysis revealed an improvement in the physical component summary score in favour of the exercise training group compared to the control group; MD 3.75 (95% CI 2.28–5.23; *p* < 0.00001) (Fig. 1b). The statistically significant improvement in the physical component summary score remained when low-quality studies were removed; MD 3.43 (95% CI 2.09–4.77; *p* < 0.00001) (Online Resources Supplemental Fig. 8). Leave-one-out sensitivity analyses did not produce any statistical changes of note.

Improvements in aerobic training; MD 4.43 (95% CI 1.02–7.83, *p* = 0.01), resistance training; MD 4.73 (95% CI 0.57–8.89, *p* = 0.03) and combined training; MD 3.78 (95% CI 2.04–5.51, *p* < 0.0001) all demonstrated statistical significance with no benefit from inspiratory muscle training, although this only represented one study (Fig. 1b). Moderate and vigorous intensity exercise, programme durations of 4–12 weeks and > 12–26 weeks, both interdialytic and intradialytic exercise, and supervised training, all demonstrated statistically significant improvements in physical component summary (Table 2, Online Resources Supplemental Figs. 9—12).

## Generic SF versus kidney disease specific QoL patient reported outcome measures

**Fig. 2:**
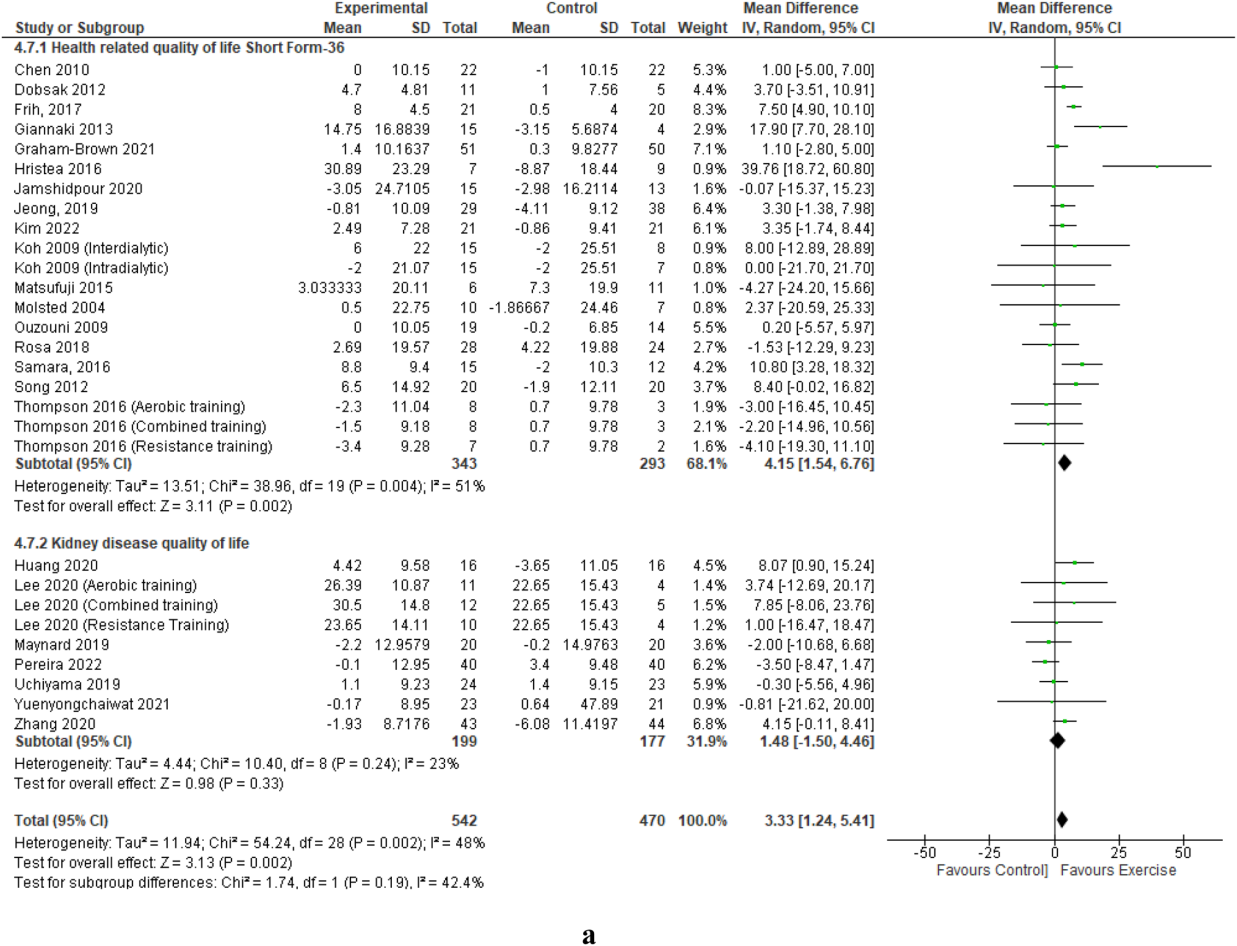

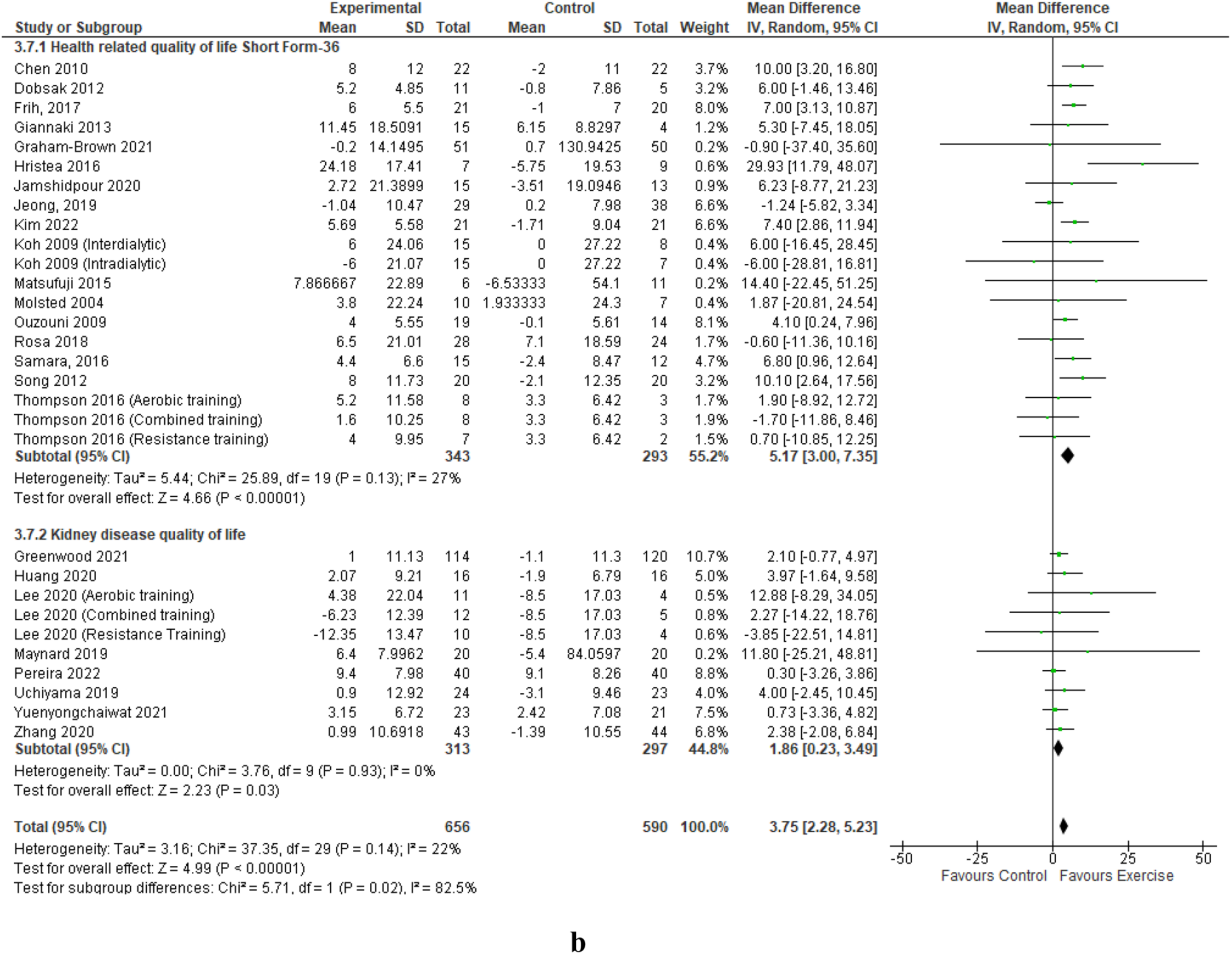
Change in Mental (**a**) and Physical (**b**) Component Summaries (score out of 100) in people with CKD stage G5 requiring dialysis with exercise as an intervention compared to a control group – Generic SF versus Kidney Disease-Specific QoL PROMs. **a** Change in Mental Component Summary – Generic SF versus Kidney Disease-Specific QoL PROMs. **b** Change in Physical Component Summary – Generic SF versus Kidney Disease-Specific QoL PROMs

Pooled data from 17 studies (20 intervention groups) that utilised the standalone generic SF QoL questionnaire demonstrated a statistically significant improvement in mental component summary; MD 4.15 (95% CI 1.54, 6.76, *p* = 0.002) compared to no significant improvement from the pooled data of 7 studies (nine intervention groups) utilising the KDQoL questionnaire (*p* = 0.33) (Fig. 2a). Both the physical component summary from the standalone SF questionnaires and from the KDQoL questionnaire resulted in significant improvements, with a larger mean point improvement from the standalone SF; 5.17 point versus 1.86-point improvement (Fig. 2b). Supplemental Figs. 13 and 14 of the Online Resources provide a sub-analysis of each type of SF and KDQoL patient-reported outcome measure utilised by the included studies.

Online Resource Supplemental Table 5 provides a brief overview of sub-analyses results revealing any statistically significant improvement in mental component summary and physical component summary in favour of exercise training.

## Heterogeneity and publication bias

Upon examination of the *I*^2^ values, we observed a moderate level of heterogeneity for the mental component summary with an *I*^2^ of 48% and a low level of heterogeneity for the physical component summary at 22%. Furthermore, our assessment using Egger funnel plots indicated minimal evidence of publication bias (Online Resources Supplemental Figs. 15 and 16).

## Study quality

When applying the RoB2 assessment tool to the outcomes of mental component summary and physical component summary, all included studies were shown to be of low risk (Online Resources Supplemental Table 6). The median TESTEX score was 11 (Online Resources Supplemental Table 7). Regarding study quality, all 25 studies clearly stated the eligibility criteria and included intervention and control groups with similar baseline data. Six of the studies did not report the randomisation details. Allocation of group was concealed to all participants until the trial began in 24 of the 25 studies, and 11 of the studies reported blinding of assessors. Concerning study reporting, adherence of over 85%, reporting of adverse effects and record of attendance at exercise sessions were fully reported by seven of the studies and one study failed to achieve any of these outcome measures. All studies reported all nominated outcomes with point estimates, eight studies performed intention-to-treat analysis, and only one study did not report between-group statistical comparisons. The exercise load was increased in all studies to maintain the intensity level required, and all included the exercise parameters, such as modality, duration, session frequency and intensity, thus enabling calculation of exercise volume and energy expenditure. Three studies [45, 47, 56] were determined to be of low quality (Online Resources Supplemental Table 7).

## Discussion

This systematic review with meta-analyses demonstrates that exercise can improve both the mental component summary and the physical component summary scores of HRQoL questionnaires in individuals with stage 5 CKD requiring dialysis. The review further indicates that an exercise training programme of aerobic or resistance training, a perceived intensity of moderate exertion, an intervention duration over 12 weeks, either inter-dialytic or intra-dialytic, and supervised training, can also positively impact the mental component summary scores. Our review also suggests that using the KDQoL patient-reported outcome measure may influence the mental component summary scores, with a mean improvement in mental component summary of 4.15 points from the SF-36 as a standalone (17 studies) versus a small and non-significant improvement of 1.48 from pooled data of 7 studies from the KDQoL; however, no direct comparison of results can be made as the patient-reported outcome measures were used in different studies. This review provides the most up-to-date analyses evaluating mental component summary and physical component summary scores by comparing exercise interventions with usual care in dialysis patients. Importantly, this review demonstrates the benefits of exercise on the mental component summary scores. Furthermore, while prior reviews have combined different QoL patient reported outcome measures, unique to this review we examined the generic SF-36 mental component summary and physical component summary scores and those of the kidney-specific SF-36 mental component summary and physical component summary scores independently.

Our findings of a positive influence of exercise training on both mental component summary and physical component summary is in contrast with recent reviews [24, 25] which identified statistical significance solely for physical component summary scores. The mean point increase in mental component summary of 3.33 was higher than that observed by Bernier-Jean in the 2022 Cochrane Review of 2.53, which was non-significant [24]. Our updated review included 24 studies for mental component summary versus 17 studies in the Cochrane Review. Leave-one-out sensitivity analysis reinforced our results, demonstrating that mental component summary and physical component summary scores were not influenced by variables such as the country of the trial, participant age, diabetes diagnoses, presence of restless legs syndrome, or the inclusion of nutritional supplements.

The correlation between poor HRQoL and mortality risk in CKD is known [70], and it has been calculated that for every 10-point decline in the score out of 100 for the mental component summary or physical component summary, the risk of mortality increases by 13% for mental component summary and 25% for physical component summary [71]. Prior studies have indicated that the inclusion of regular exercise as an additional form of treatment has the potential to reduce mortality when there is an increase in QoL scores, as well as improve patients’ ability to perform daily tasks and reduce the burden of dialysis [71]. The overall finding that exercise training improved the mental component summary score aligns with the positive findings from reviews on the effect of exercise on depression in haemodialysis patients [24, 72]. Depression can have a detrimental effect on QoL in people with CKD. While the SF-36 is not defined as a depression patient-reported outcome measure, it does assess mental health comprising of questions associated with vitality, social functioning, role emotional and mental health. Findings of our review underscore the importance of incorporating exercise training into the treatment regimen for individuals on dialysis to assist in improving the mental health and wellbeing of dialysis patients.

It has been reported that a change in mental component summary or physical component summary greater than or equal to five is considered clinically important [73]. In those studies that used the HRQoL standalone SF-36 questionnaire, sub-analysis showed that both the mental component summary and physical component summary scores improved significantly with exercise training, reaching a mean 5.17 point improvement for physical component summary, and just missing this five-point improvement for mental component summary (4.15 points). Furthermore, several of the results examining modality approached this five-point mark, with a mean improvement of 4.57 points in mental component summary and 4.43 points in physical component summary observed from aerobic training, and a 4.73-point improvement in physical component summary from resistance training. Notably, the sub-analysis of studies employing the KDQoL questionnaire did not reveal a statistically significant improvement in the mental component summary scores and only improved by a mean of 1.86 points for physical component summary. This may indicate that despite containing the same questions as the SF-36 at its generic core, the additional disease-specific questions in the KDQoL may influence participants to respond differently to the SF-36 questions compared to those completing the SF-36 as a standalone patient reported outcome measure, resulting in a lower score. Clinicians should consider this when analysing results and deciding whether to include or continue exercise training in an individual’s treatment regimen.

Overall, sub-analysis revealed that engaging in aerobic, resistance or a combination of both modalities can improve physical component summary scores, with aerobic and resistance improving mental component summary, and a trend for mental component summary improvement with combined training. These results contrast with those of the review by Bernier-Jean (2022) [24] who found only aerobic and combined exercise training positively impacted physical component summary, and Hu (2022) [25], who, while also finding that all three modalities positively impacted physical component summary, reported that only resistance training enhanced mental component summary. These findings are promising as they indicate the efficacy of a broad range of exercise modalities. Consequently, participants have the flexibility to select an exercise modality that aligns with their personal preferences and capabilities.

Analysis of exercise intensity revealed that participants who engaged in an exercise programme targeting a perceived ‘moderate’ exertion level experienced improvements to both mental component summary and physical component summary scores. Conversely, participants restricted to a ‘light’ intensity programme did not show an improvement in the physical component summary score, while those assigned to a ‘vigorous’ intensity programme did not exhibit improvements in mental component summary scores. These findings align with intuitive expectations: light-intensity exercise is likely to have minimal effect on physical functioning, whereas vigorous-intensity exercise may be emotionally challenging, particularly when compounded by the usual stresses of living with CKD. Therefore, these results are not surprising and underscore the importance of tailoring exercise intensity to achieve benefits for individuals requiring dialysis.

Intervention duration indicated that exercise training up to 12 weeks resulted in an improvement in physical component summary scores compared to the usual care control group. Notably, both mental component summary and physical component summary scores were enhanced in trials lasting between 12 and 26 weeks. These findings suggest that to achieve improvements in both mental component summary and physical component summary scores, the exercise programme should extend beyond 12 weeks. Additionally, our results indicated that exercise durations exceeding 26 weeks did not produce significant positive effects on physical component summary or mental component summary scores when compared to the control group. However, it is important to note that the sub-analysis of greater than 26 weeks was based on data from only two studies, and therefore these findings should be interpreted with caution.

Exercise performed either during dialysis sessions (intra-dialytic) or outside of dialysis sessions (inter-dialytic) both had a positive impact on mental component summary and physical component summary scores. This finding is particularly important for individuals with stage 5 CKD, as it suggests that regular exercise can lead to improvements in mental component summary and physical component summary scores regardless of the availability of exercise equipment within the dialysis unit or the participant’s ability to exercise at specific times. Only supervised exercise sessions were successful in improving mental component summary and physical component summary scores. Supervised exercise programmes often lead to better adherence, motivation and overall effectiveness of the exercise session [74]. The Hawthorne effect, where participants alter their behaviour due to the awareness of being observed, should be considered as having a contributing effect to our results [75]. This phenomenon suggests that the noted improvements in mental component summary and physical component summary scores might not be solely attributable to exercise training itself, but also to the increased attention and supervision received by the participants. Supervised training also provides participants with increased social engagement, a key component in mental health and wellbeing, which likely further leads to improvements. Additionally, it is important to once again note that only two of our included trials involved unsupervised exercise sessions. Further trials of unsupervised exercise programmes involving participants with stage 5 CDK may be necessary to determine whether they can also lead to improvements in mental component summary and physical component summary scores when compared to a control group of no exercise.

We consider this systematic review to be the most thorough investigation of the effect of exercise on mental component summary and physical component summary scores in individuals living with stage 5 CKD to date, with minimal evidence of publication bias. However, it is important to recognise several limitations within our review. Measurements of HRQoL using patient reported outcome measures are subjective and dynamic which can lead to variability in participants’ answers to each question both at baseline and completion of the trial [76] ultimately leading to a possible impact on mental component summary and physical component summary scores. Floor and ceiling effects can occur when using patient reported outcome measures. If a substantial proportion of participants record the highest or lowest score possible, it becomes difficult to discriminate between respondents at either end of the scale and to detect change in the measure over time, thus leading to misinterpretation of the data or bias. Despite the leave-one-out sensitivity analysis indicating no trial influenced the results, factors such as mood and current health status at the time of filling in the questionnaire and cultural differences, including language and how individuals perceive and report their QoL may have impacted the reliability and validity of the data collected. The utilisation of different questionnaires may have also impacted the results, despite this meta-analysis limiting included studies to those utilising versions of the SF-36 only. Additionally, there are numerous ways to calculate the mental component summary and physical component summary scores, which may have altered the final scores [77].

Exclusion of participants based on comorbidities may have introduced bias. Participants included in the intervention, particularly those supervised during their exercise session, often exhibit higher motivation to perform exercises consistently and to a high standard, compared to those training unsupervised, potentially introducing bias. Additionally, the diversity of exercise modalities, variations in trial programmes (including duration and frequency), and subjective perceptions of intensity could have influenced the scores. Setting a definitive time for resistance training is challenging, as some participants exercise faster while others require longer recovery periods between sets or repetitions and these differences may also lead to bias.

Statistically, there is a potential for bias when subtracting the baseline mean from the final mean value, as participants with lower baseline fitness levels may exhibit more pronounced improvements when compared to those who began with a reasonable to high fitness level. Blinding of the participants is not possible in exercise-based trials, and few studies blinded the assessors, these factors could have also influenced the quality of the data.

Our review indicates that exercise training can improve mental component summary and physical component summary scores in individuals with CKD on dialysis. However, the longer-term impact of exercise on HRQoL remains unknown. Future studies of longer duration are needed to determine the sustained effects of exercise training on QoL. Additionally, few trials have included an exercise training programme designed to achieve perceived intensity/exertion rates of either light or vigorous levels. Future trials focusing on these specific intensities may uncover additional health benefits, potentially leading to greater enhancements in mental component summary and physical component summary scores.

## Conclusions

Our analysis indicated that the inclusion of supervised, inter-dialytic or intra-dialytic exercise of either aerobic or resistance training, a perception of a ‘moderate’ intensity, for a period of 12–26 weeks can improve both mental component summary and physical component summary scores in people with stage 5 CKD.

## Supplementary Information

Below is the link to the electronic supplementary material.

Supplementary file1 (DOCX 871 KB)

## Data Availability

Data for this analysis is available from the corresponding author.

## Abbreviations

CKD: Chronic kidney disease
ExTr: Exercise training
HRQoL: Health related quality of life
KDQoL: Kidney disease quality of life
MCS: Mental component summary
PCS: Physical component summary
PROMs: Patient-reported outcome measure questionnaires
QoL: Quality of life
RCT: Randomised controlled trial
SF: Short-form
