# Supplementary material for: The effect of exercise training on quality of life in people with chronic kidney disease requiring dialysis. A systematic review with meta-analysis
Source: J Nephrol. 2025 Mar 28;38(3):893–911. doi: 10.1007/s40620-025-02245-1 (PMC12165990; doi:10.1007/s40620-025-02245-1)
Supplement: Supplementary file 1 — Supplementary file1 (DOCX 871 KB) [file 40620_2025_2245_MOESM1_ESM.docx]

**The effect of exercise training on quality of life in people with chronic kidney disease requiring dialysis. A systematic review with meta-analysis.**

Annette Traise*, Gudrun Dieberg, Elizabeth Degotardi, Bailey Hart, Fiza Kaippilly, Darcy McInnes, Melissa J Pearson, David Ryan, Neil A Smart

Clinical Exercise Physiology, School of Science and Technology, University of New England, NSW 2351, Australia

* Corresponding author

# **Online Resources**

## Table of Content

| **Supplemental Figures** |  |
| --- | --- |
| **Supplemental Figure 1** Details of commonalities and relationship of health-related quality of life questionnaires | Page 3 |
| **Supplemental Figure 2** PRISMA flow diagram | Page 4 |
| **Supplemental Figure 3** Change in Mental component summary (score out of 100) in people with CKD stage 5 requiring dialysis with exercise as an intervention compared to a control group – by exercise modality with low quality studies removed | Page 5 |
| **Supplemental Figure 4** Change in Mental Component Summary (score out of 100) in people with CKD stage G5 requiring dialysis with exercise as an intervention compared to a control group – by exercise intensity | Page 6 |
| **Supplemental Figure 5** Change in Mental Component Summary (score out of 100) in people with CKD stage G5 requiring dialysis with exercise as an intervention compared to a control group – by intervention duration | Page 7 |
| **Supplemental Figure 6** Change in Mental Component Summary (score out of 100) in people with CKD stage G5 requiring dialysis with exercise as an intervention compared to a control group – by timing of intervention (interdialytic and intradialytic) | Page 8 |
| **Supplemental Figure 7** Change in Mental Component Summary (score out of 100) in people with CKD stage G5 requiring dialysis with exercise as an intervention compared to a control group – by intervention supervision and non-supervision | Page 9 |
| **Supplemental Figure 8** Change in Physical component summary (score out of 100) in people with CKD stage 5 requiring dialysis with exercise as an intervention compared to a control group – by exercise modality with low quality studies removed | Page 10 |
| **Supplemental Figure 9** Change in Physical Component Summary (score out of 100) in people with CKD stage G5 requiring dialysis with exercise as an intervention compared to a control group – by exercise intensity | Page 11 |
| **Supplemental Figure 10** Change in Physical Component Summary (score out of 100) in people with CKD stage G5 requiring dialysis with exercise as an intervention compared to a control group – by intervention duration | Page 12 |
| **Supplemental Figure 11** Change in Physical Component Summary (score out of 100) in people with CKD stage G5 requiring dialysis with exercise as an intervention compared to a control group – by timing of intervention (interdialytic and intradialytic) | Page 13 |
| **Supplemental Figure 12** Change in Physical Component Summary (score out of 100) in people with CKD stage G5 requiring dialysis with exercise as an intervention compared to a control group – by intervention supervision and non-supervision | Page 14 |
| **Supplemental Figure 13** Change in Mental Component Summary (score out of 100) in people with CKD stage G5 requiring dialysis with exercise as an intervention compared to a control group – by type of health questionnaire used | Page 15 |
| **Supplemental Figure 14** Change in Physical Component Summary (score out of 100) in people with CKD stage G5 requiring dialysis with exercise as an intervention compared to a control group – by type of health questionnaire used | Page 16 |
| **Supplemental Figure 15** Egger Funnel Plot of Comparison Mental Component Summary (score out of 100) | Page 17 |
| **Supplemental Figure 16** Egger Funnel Plot of Comparison Physical Component Summary (score out of 100) | Page 18 |
| **Supplemental Tables** |  |
| **Supplemental Table 1** Search strategy and results | Page 19 |
| **Supplemental Table 2** Intensity sub-analysis categories based on the BORG rating of perceived exertion | Page 20 |
| **Supplemental Table 3** Excluded studies | Page 21 |
| **Supplemental Table 4** Detailed Exercise Intervention Characteristics | Page 23 |
| **Supplemental Table 5** Brief summary of sub-analyses indicating if exercise led to a significant improvement in outcomes in favour of the intervention group | Page 27 |
| **Supplemental Table 6** Cochrane risk of bias tool for randomised controlled trials (Rob2) | Page 28 |
| **Supplemental Table 7** Assessment of study quality and reporting using Tool for the assessment of study quality and reporting in exercise (TESTEX) | Page 30 |
| **References** | Page 31 |

**Supplemental Figure 1** Details of commonalities and relationship of health-related quality of life questionnaires.

**Supplemental Figure 2** PRISMA flow diagram


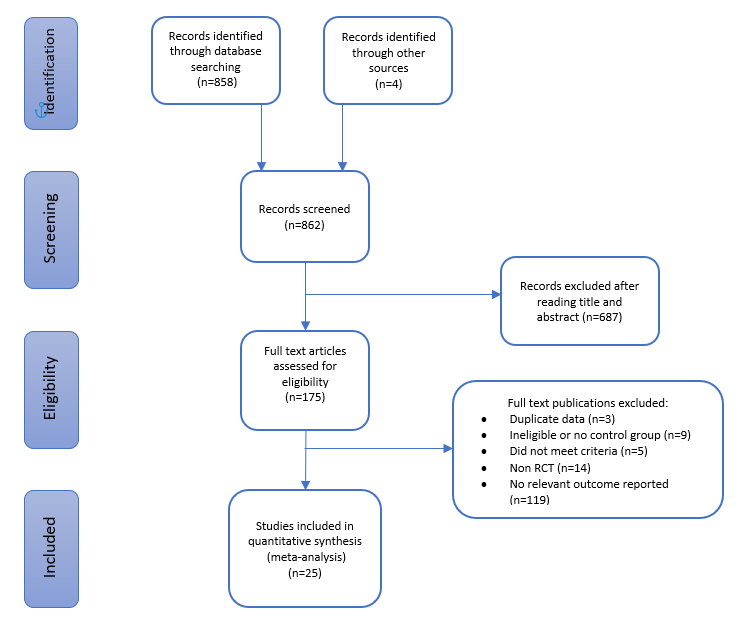


**Supplemental Figure 3** Change in Mental Component Summary (score out of 100) in people with CKD stage 5 requiring dialysis with exercise as an intervention compared to a control group – by exercise modality with low quality studies removed (p=0.002 when all studies included)


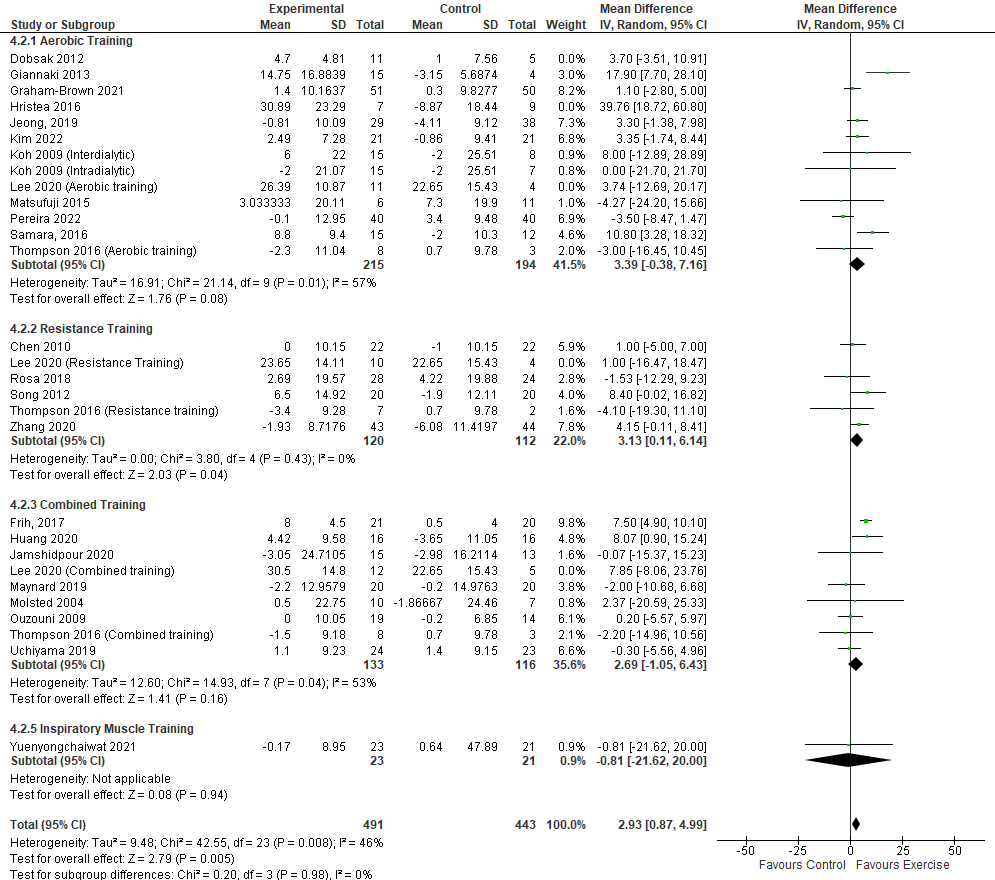


**Supplemental Figure 4** Change in Mental Component Summary (score out of 100) in people with CKD stage G5 requiring dialysis with exercise as an intervention compared to a control group – by exercise intensity


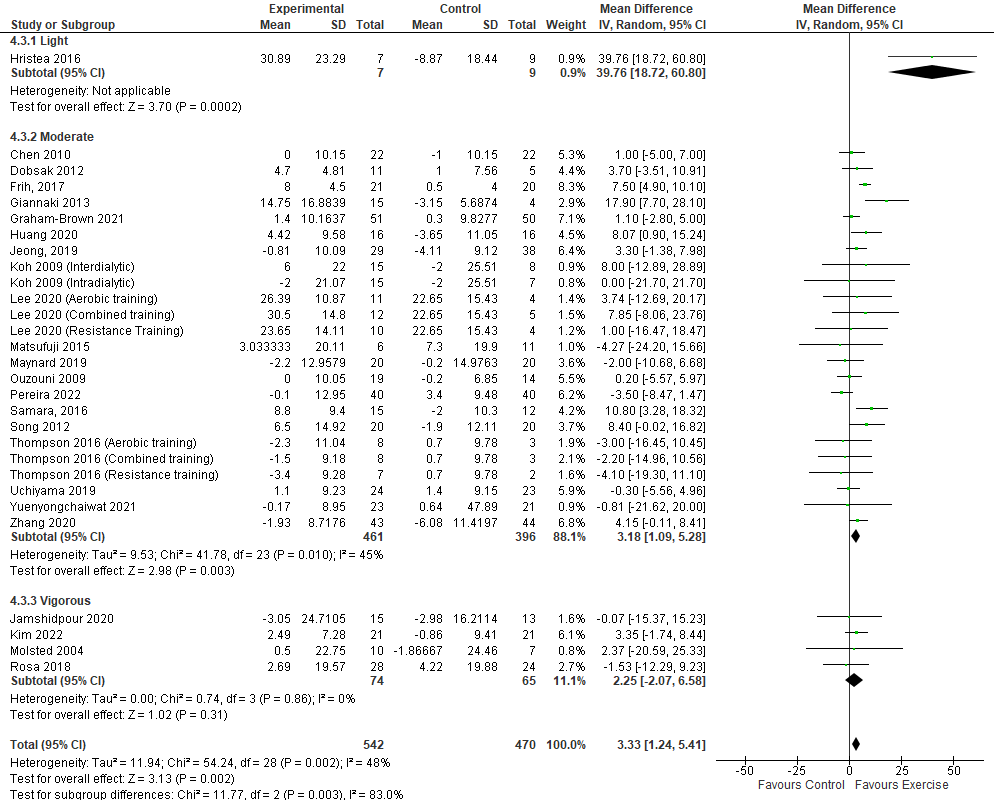


**Supplemental Figure 5** Change in Mental Component Summary (score out of 100) in people with CKD stage G5 requiring dialysis with exercise as an intervention compared to a control group – by intervention duration

**
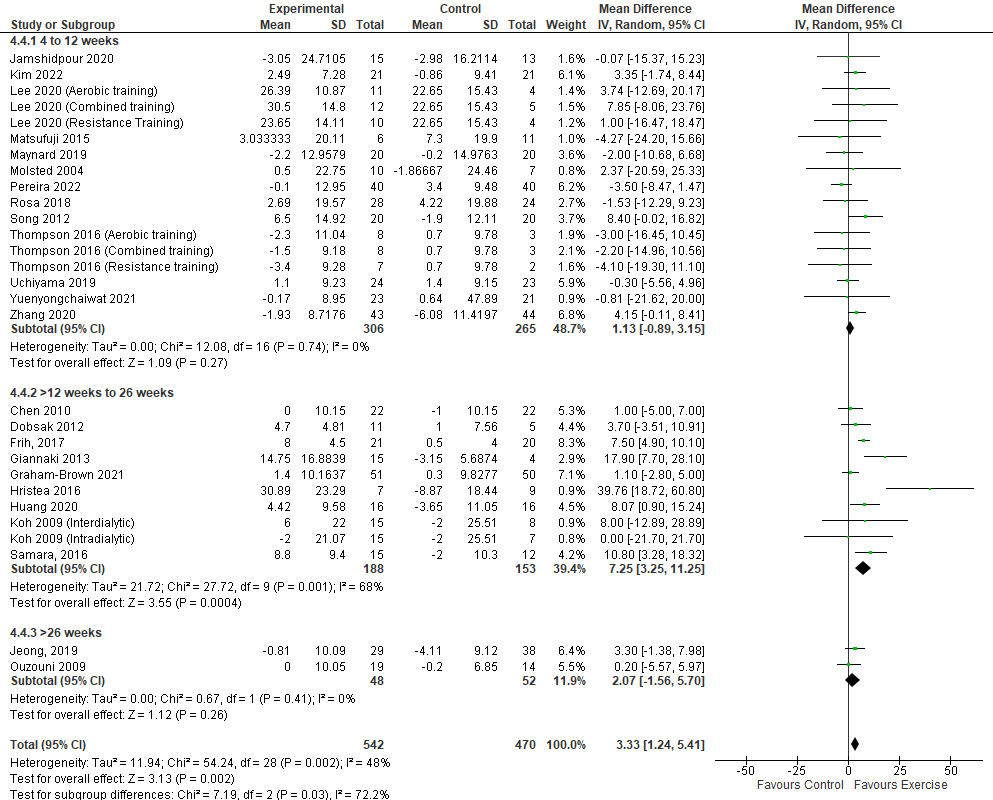
**

**Supplemental Figure 6** Change in Mental Component Summary (score out of 100) in people with CKD stage G5 requiring dialysis with exercise as an intervention compared to a control group – by timing of intervention (interdialytic and intradialytic)

**
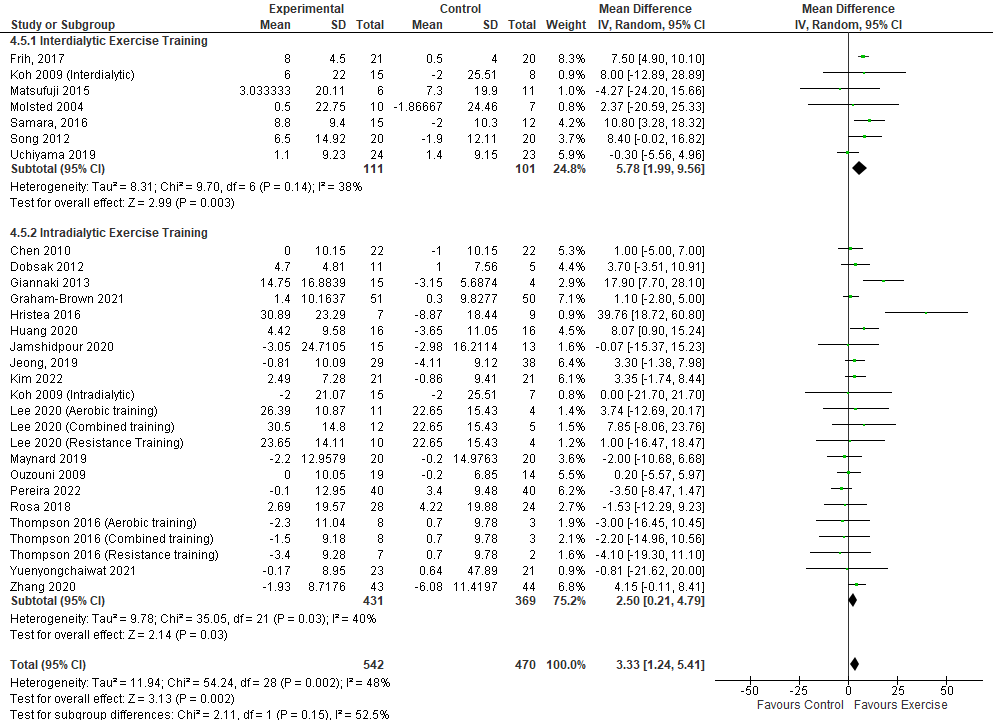
**

**Supplemental Figure 7** Change in Mental Component Summary (score out of 100) in people with CKD stage G5 requiring dialysis with exercise as an intervention compared to a control group – by intervention supervision and non-supervision


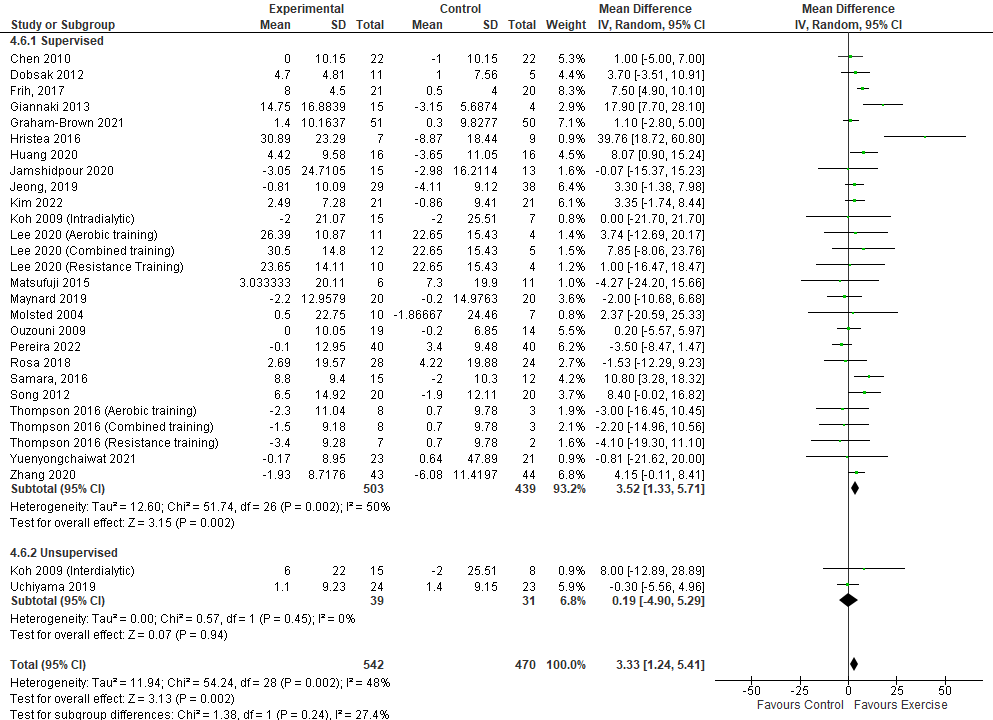


**Supplemental Figure 8** Change in Physical Component Summary (score out of 100) in people with CKD stage 5 requiring dialysis with exercise as an intervention compared to a control group – by exercise modality with low quality studies removed (p<0.00001 when all studies included)


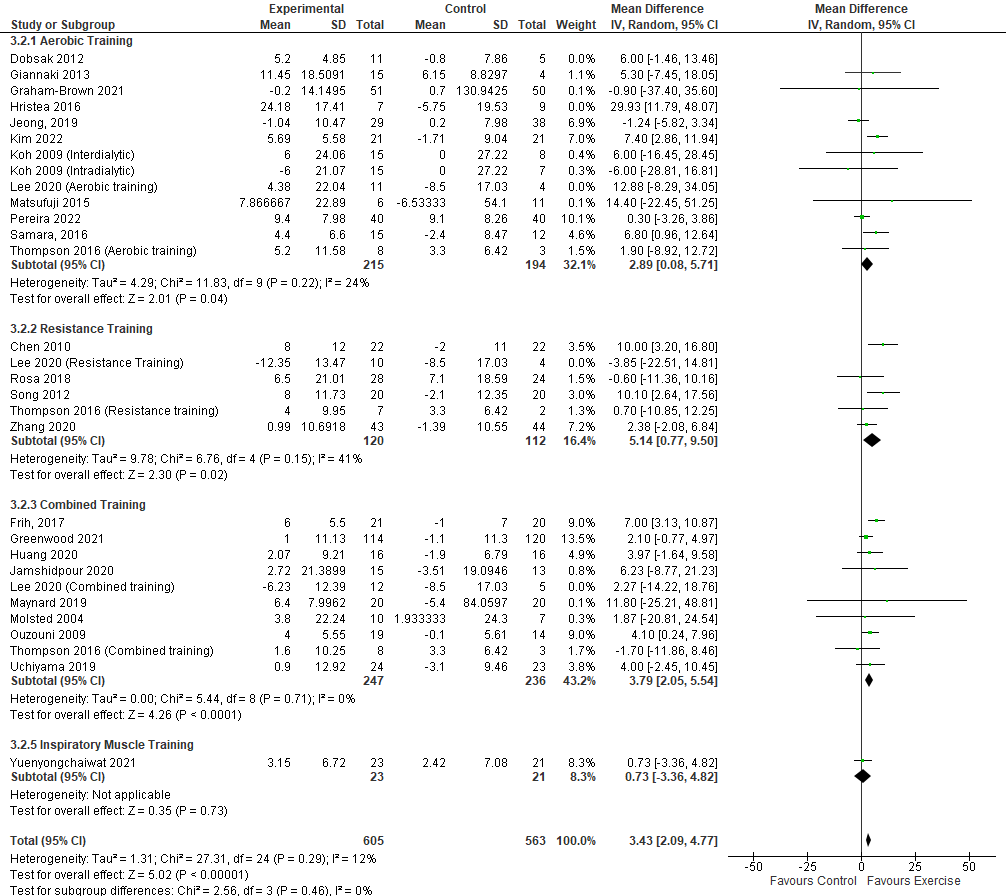


**Supplemental Figure 9** Change in Physical Component Summary (score out of 100) in people with CKD stage G5 requiring dialysis with exercise as an intervention compared to a control group – by exercise intensity


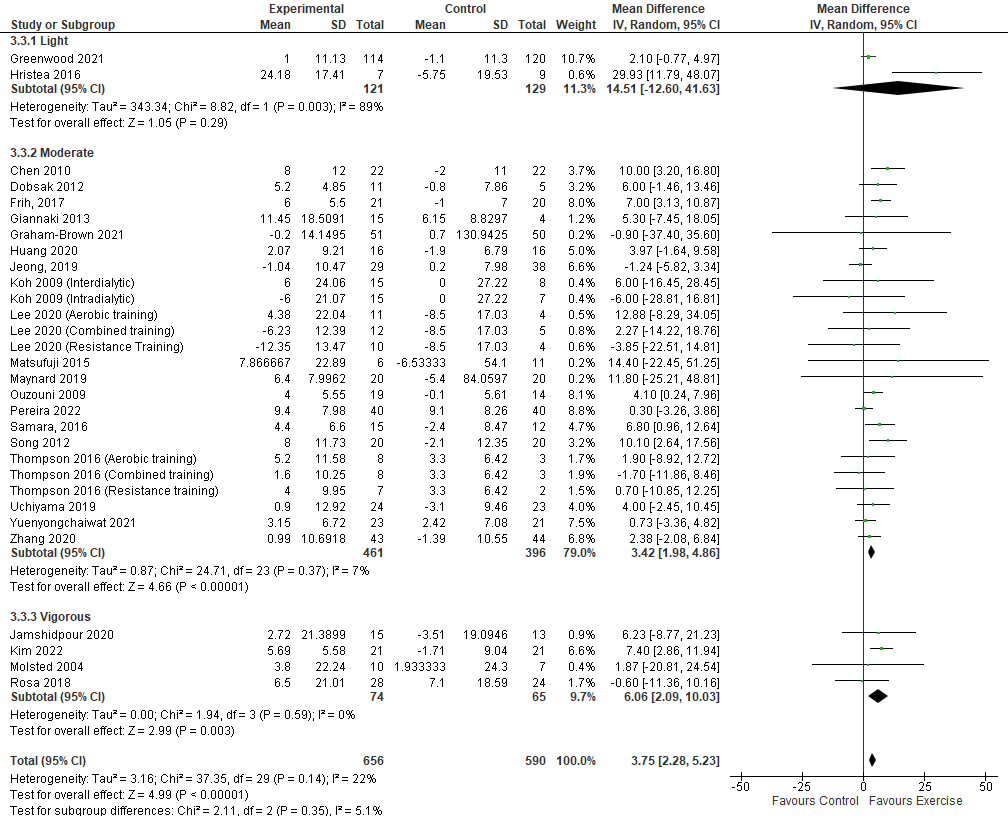


**Supplemental Figure 10** Change in Physical Component Summary (score out of 100) in people with CKD stage G5 requiring dialysis with exercise as an intervention compared to a control group – by intervention duration


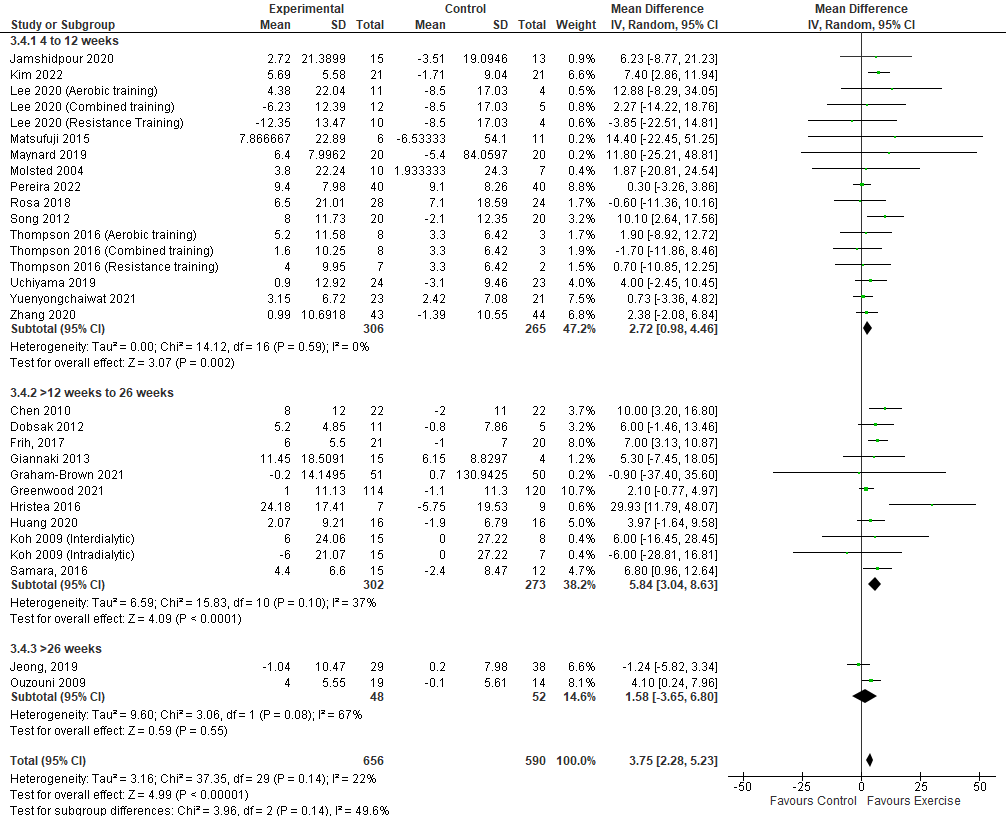


**Supplemental Figure 11** Change in Physical Component Summary (score out of 100) in people with CKD stage G5 requiring dialysis with exercise as an intervention compared to a control group – by timing of intervention (interdialytic and intradialytic)


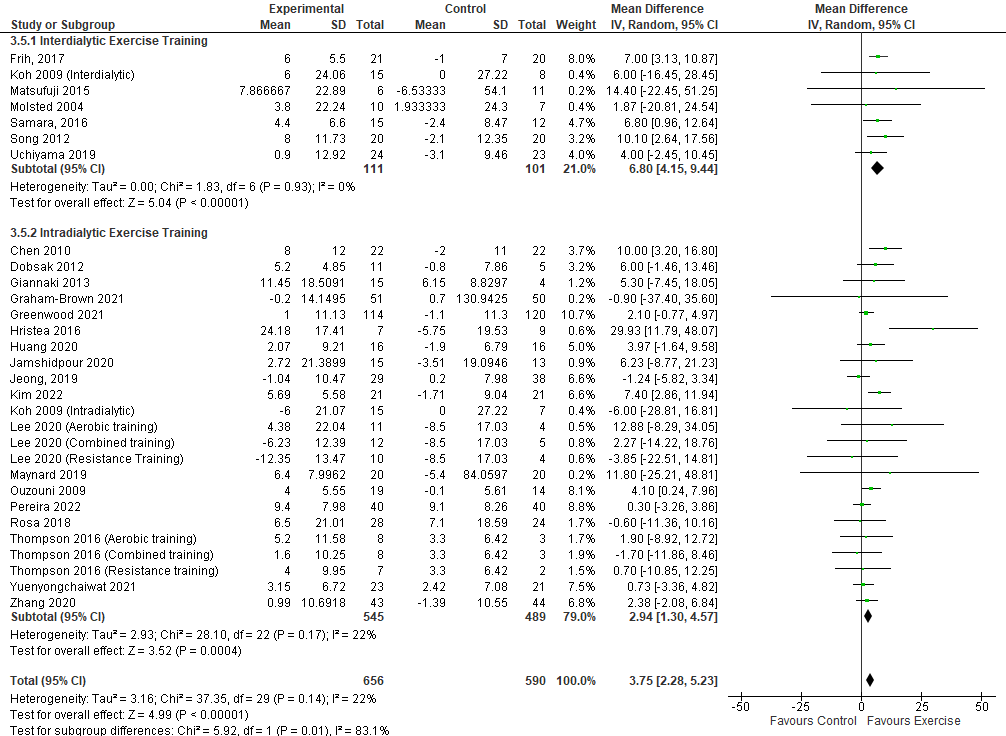


**Supplemental Figure 12** Change in Physical Component Summary (score out of 100) in people with CKD stage G5 requiring dialysis with exercise as an intervention compared to a control group – by intervention supervision and non-supervision


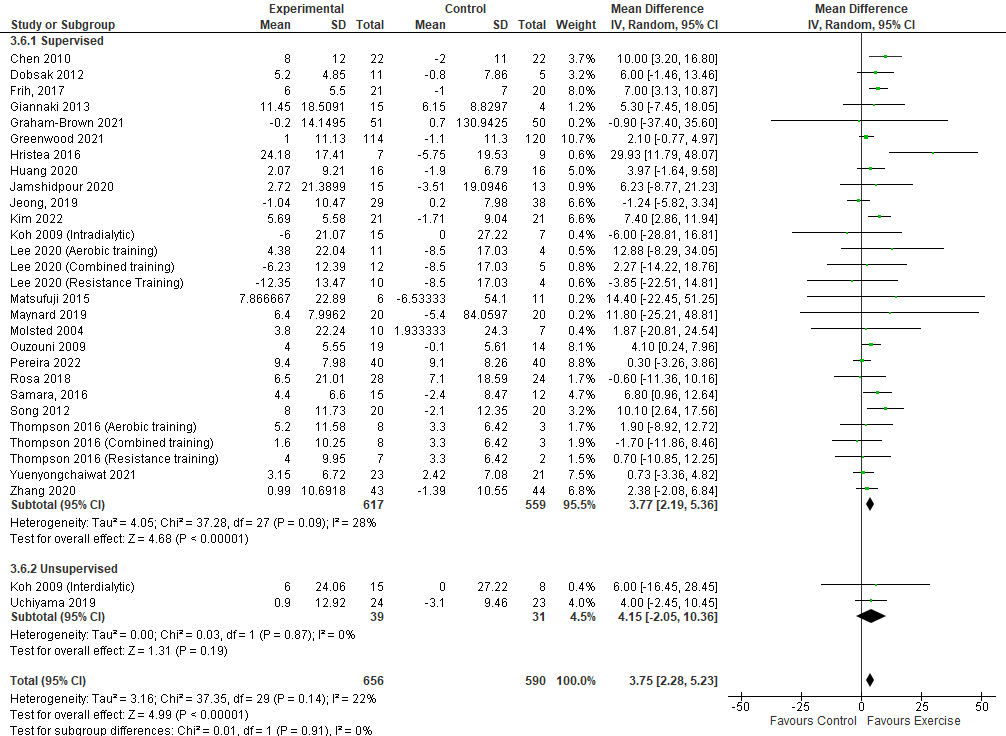


**Supplemental Figure 13** Change in Mental Component Summary (score out of 100) in people with CKD stage G5 requiring dialysis with exercise as an intervention compared to a control group – by type of health questionnaire used


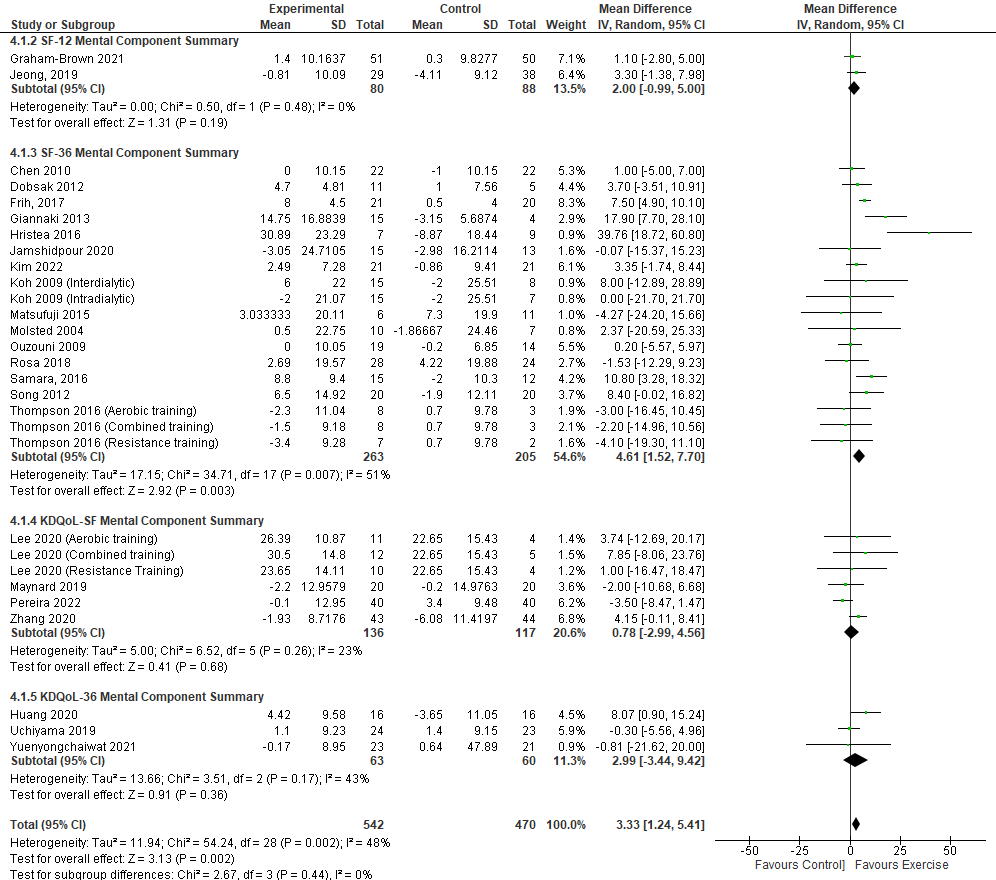


**Supplemental Figure 14** Change in Physical Component Summary (score out of 100) in people with CKD stage G5 requiring dialysis with exercise as an intervention compared to a control group – by type of health questionnaire used


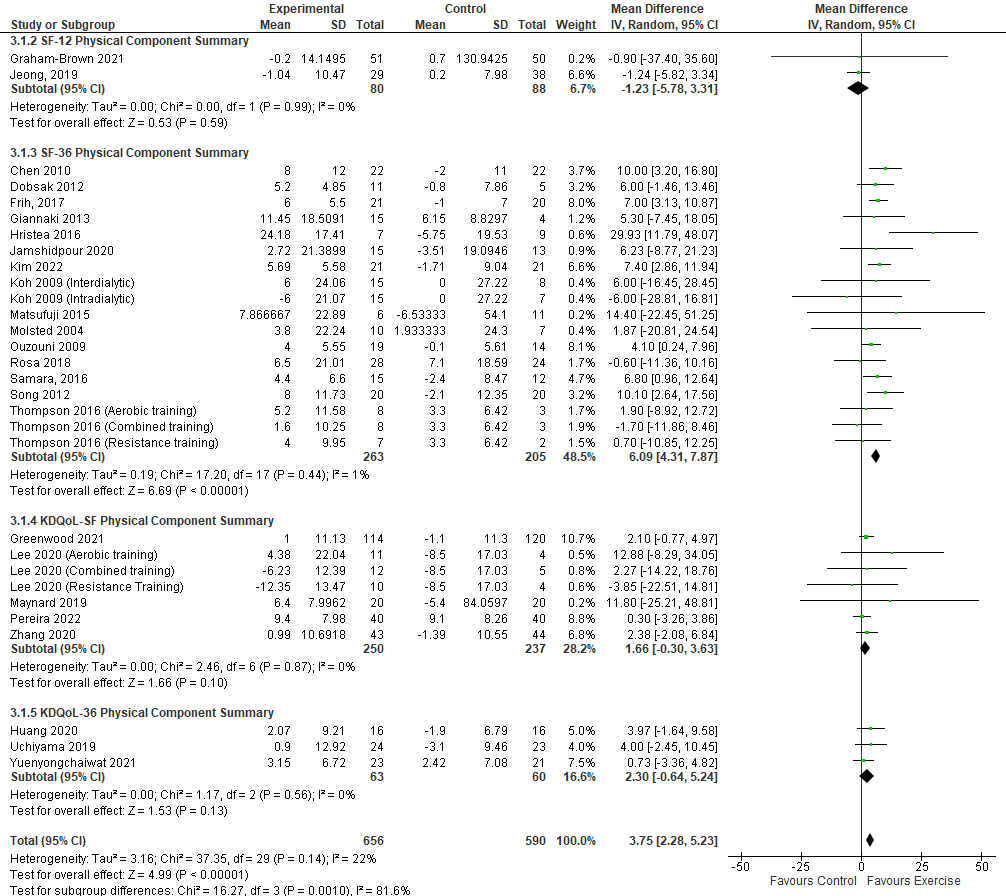


**Supplemental Figure 15** Egger Funnel Plot of Comparison Mental Component Summary (MCS) (score out of 100) p=0.002

**SF15a**: MCS by intervention modality; **SF15b**: MCS by intervention intensity; **SF15c**: MCS by intervention duration; **SF15d**: MCS by intervention timing; **SF15e**: MCS by intervention supervision; **SF15f**: MCS by intervention health questionnaire

| **SF15a:** MCS by modality  **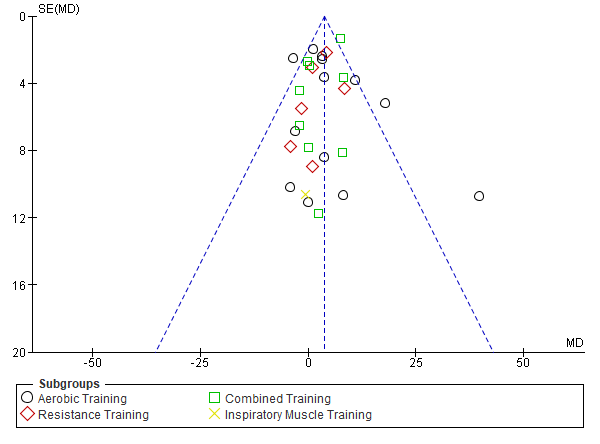** | **SF15b**: MCS by intensity  **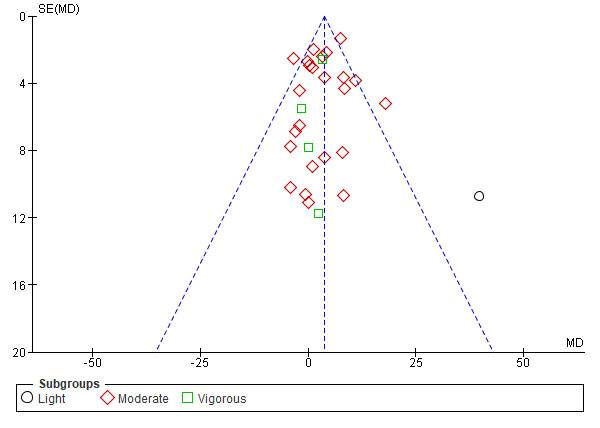** |
| --- | --- |
| **SF15c**: MCS by duration  **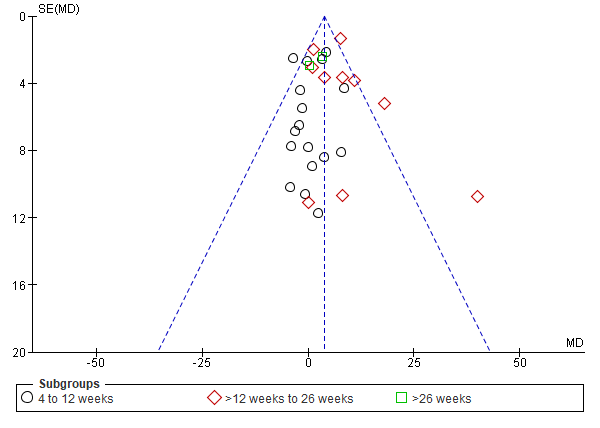** | **SF15d**: MCS by timing  **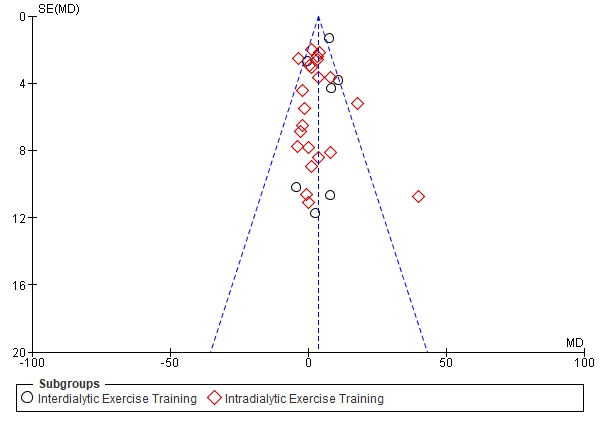** |
| **SF15f** MCS by supervision  **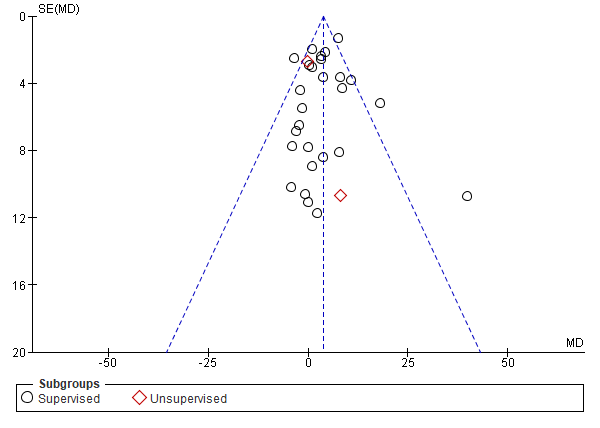** | **SF15e:** MCS by questionnaire **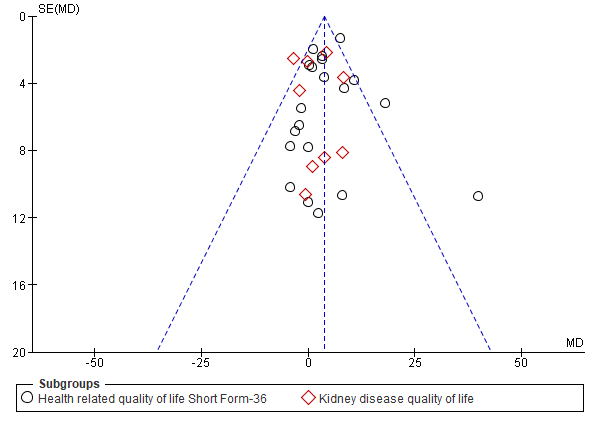** |

**Supplemental Figure 16** Egger Funnel Plot of Comparison Physical Component Summary (PCS) (score out of 100) p<0.00001

**SF16a**: PCS by intervention modality; **SF16b**: PCS by intervention intensity; **SF16c**: PCS by intervention duration; **SF16d**: PCS by intervention timing; **SF16e**: PCS by intervention supervision;

| **SF16a:** PCS by modality  **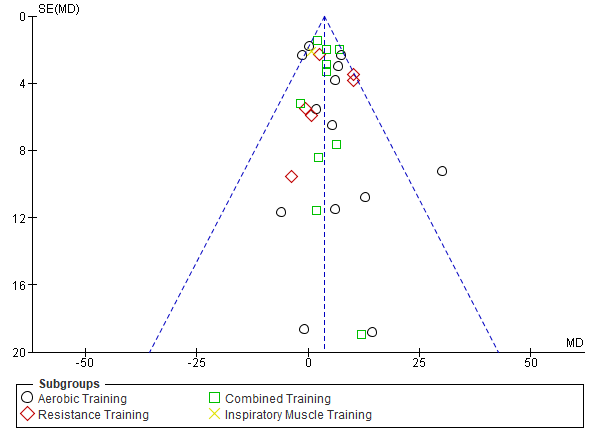** | **SF16b**: PCS by intensity  **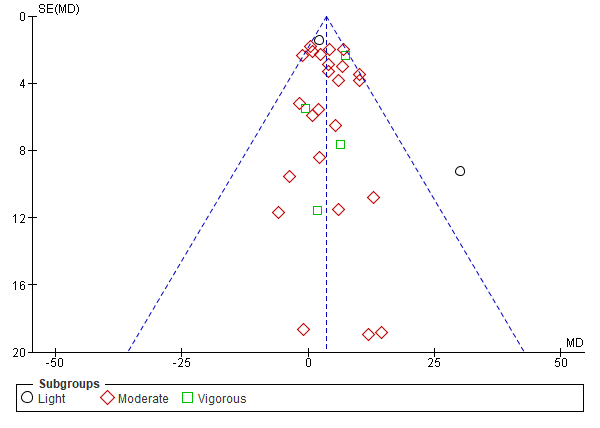** |
| --- | --- |
| **SF16c**: PCS by duration  **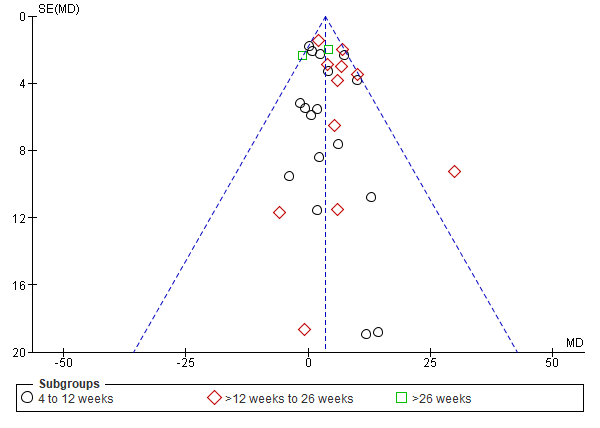** | **SF16d**: PCS by timing  **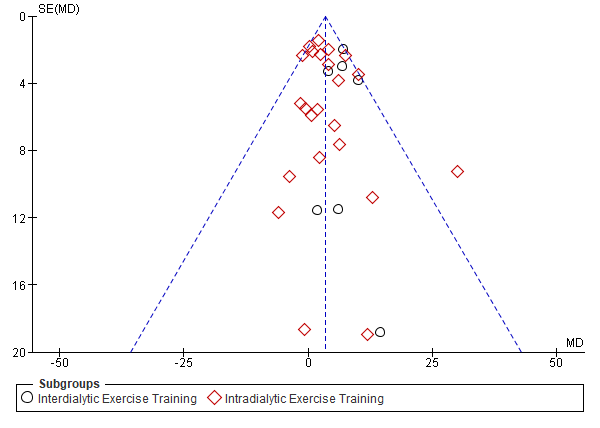** |
| **SF16e**: PCS by supervision  **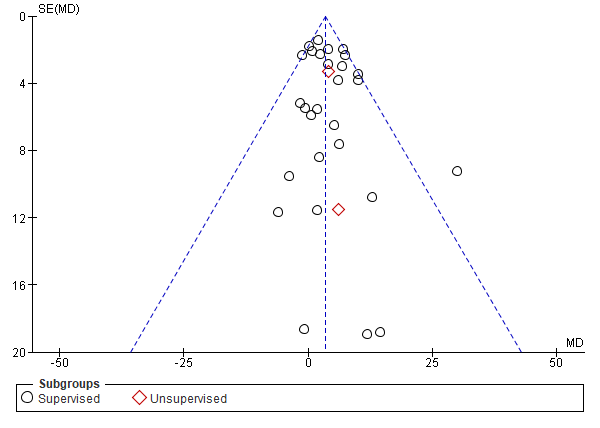** | **SF16f:** PCS by questionnaire  **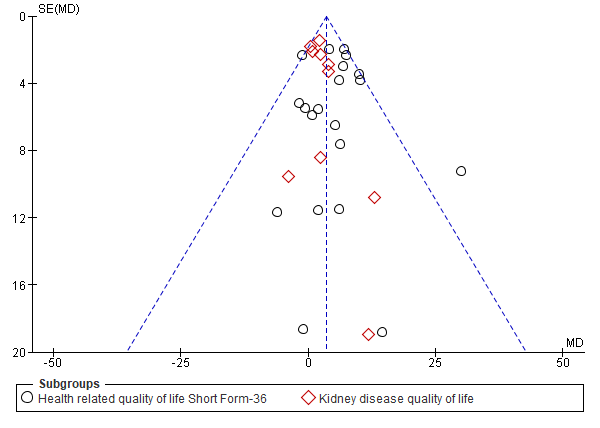** |

**Supplemental Table 1:** Search strategy and results

| **#** | **MEDLINE ALL Query** | **Results 14^th^ November 2024** |
| --- | --- | --- |
| 1 | (Chronic kidney disease or renal disease or CKD).mp. | 117,978 |
| 2 | (exercise or physical activit* or fitness).mp. | 507,508 |
| 3 | #1 and #2 | 2,366 |
| 4 | limit #3 to randomized controlled trial | 240 |
|  | **EMBASE Query** | **Results 14^th^ November 2024** |
| 1 | ((chronic AND kidney AND disease OR chronic) AND renal AND disease OR chronic) AND renal AND failure | 183,755 |
| 2 | (exercise OR physical) AND activit* OR fitness | 794,986 |
| 3 | #1 AND #2 | 2,689 |
| 4 | #3 AND 'randomized controlled trial'/de | 17 |
| **#** | **Cochrane Library of Controlled Trials Query** | **Results 14^th^ November 2024** |
| 1 | (Chronic kidney disease or renal disease or CKD):ti,ab,kw | 17,828 |
| 2 | #1 AND Exercise or physical activit* or fitness | 1, 058 |
| 3 | #2 AND randomised controlled trial | 446 |
| 4 | #3 NOT pre-dialysis OR predialysis | 330 |
|  | **CINAHL Query** | **Results 14^th^ November 2024** |
| 1 | kidney disease or chronic renal failure or ckd or esrd or renal insufficiency or kidney failure | 74,682 |
| 2 | #1 AND exercise or 'physical activity' or fitness or walking | 2,116 |
| 3 | #2 AND randomized controlled trials or rtc or randomised control trials | 232 |
|  | **SPORTDiscus Query** | **Results 14^th^ November 2024** |
| 1 | (“chronic kidney disease” or “chronic renal failure” or ckd) | 1,421 |
| 2 | #1 AND (exercise or “physical activity” or fitness) | 229 |
| 3 | #2 AND (“randomized controlled trials” or rtc or “randomised control trials”) | 31 |

**Supplemental Table 2:** Intensity sub-analysis categories based on the BORG rating of perceived exertion

| Category | BORG scale 0-10 and Description | | BORG scale 6-20 and Description | |
| --- | --- | --- | --- | --- |
| Light | 0  0.5  1  2 | Nothing at all  Very, very slight  Very slight  Slight | 6  7.5  9  11 | No exertion at all  Extremely light  Very light  Light |
| Moderate | 3  4 | Moderate  Somewhat severe | 13 | Somewhat hard |
| Vigorous | 5  7  9  10 | Severe  Very severe  Very, very severe  Maximal | 15  17  19  20 | Hard  Very hard  Extremely hard  Maximal exertion |

**Supplemental Table 3:** Excluded studies

| **Study Author, year** | **Reason for exclusion** | **Study Author, year** | **Reason for exclusion** | **Study Author, year** | **Reason for exclusion** |
| --- | --- | --- | --- | --- | --- |
| Abdelbasset, 2022 | No relevant outcome data | García Testal, 2019 | No relevant outcome data | Oliveira, 2019 | No relevant outcome data |
| Abreu, 2017 | No relevant outcome data | Goldberg, 1983 | No relevant outcome data | Olvera-Soto, 2016 | No relevant outcome data |
| Afshar, 2011 | No relevant outcome data | Greenwood, 2024 | Did not meet criteria | Paglialonga, 2014 | Did not meet criteria |
| Amirtha, 2018 | No relevant outcome data | Groussard, 2014 | No relevant outcome data | Painter, 2012 | No relevant outcome data |
| Anding, 2015 | No relevant outcome data | Hamada, 2016 | Not RCT | Paluchamy, 2018 | No relevant outcome data |
| Andrade, 2021 | No relevant outcome data | Harter, 1985 | Duplicate Goldberg, 1983 | Pandey, 2017 | No relevant outcome data |
| Assawasakskul, 2021 | No relevant outcome data | Highton, 2021 | No relevant outcome data | Papal, 2022 | No relevant outcome data |
| Bagetta, 2018 | Duplicate Manfredini, 2017 | Homma, 2022 | No relevant outcome data | Parsons, 2004 | No relevant outcome data |
| Basir, 2022 | No relevant outcome data | Hornik, 2021 | No relevant outcome data | Dipp, 2018 | No relevant outcome data |
| Bastani, 2020 | No relevant outcome data | Dipp, 2018 | No relevant outcome data | Pellizaro, 2013 | No relevant outcome data |
| Belik, 2018 | No relevant outcome data | Huang, 2021 | No relevant outcome data | Perez-Dominguez, 2021 | No control group |
| Bennett, 2006 | Not RCT | Ibrahim, 2021 | No relevant outcome data | Petraki, 2008 | No relevant outcome data |
| Bennett, 2016 | No control group | Jang, 2009 | Not RCT | Pomidori, 2016 | No relevant outcome data |
| Brito, 2022 | No relevant outcome data | Jaramillo-Morales, 2022 | No relevant outcome data | Poorsaadet, 2018 | No relevant outcome data |
| Brown, 2018 | No relevant outcome data | Jeong, 2023 | No relevant outcome data | Pugh-Clarke, 2002 | Not RCT |
| Campos, 2018 | No relevant outcome data | Johansen, 2006 | No relevant outcome data | Rahimimoghadam, 2019 | No control group |
| Cappy, 1999 | Not RCT | Johnstone, 2002 | Not RCT | Rezaei, 2014 | No relevant outcome data |
| Careless, 2017 | No relevant outcome data | Konstantinou, 2002 | No relevant outcome data | Reboredo, 2014 | No relevant outcome data |
| Carmack, 1995 | No relevant outcome data | Kopple, 2007 | No relevant outcome data | Rhee, 2019 | Not RCT |
| Cardosa, 2020 | No relevant outcome data | Koufaki, 2002 | No relevant outcome data | Roxo, 2016 | No relevant outcome data |
| Carney, 1987 | No relevant outcome data | Kouidi, 1997 | No relevant outcome data | Sakkas , 2008 | Not RCT |
| Chan, 2016 | Not RCT | Kouidi, 2009 | No relevant outcome data | Salehi, 2022 | No relevant outcome data |
| Cheema, 2007 | No relevant outcome data | Kumar, 2022 | No relevant outcome data | Santhi, 2018 | No relevant outcome data |
| Cheema, 2011 | No relevant outcome data | Krase, 2021 | No relevant outcome data | Sarmento, 2017 | No control group |
| Cho, 2017 | No relevant outcome data | Liao, 2016 | No relevant outcome data | Segura-Orti, 2009 | No control group |
| Clarkson, 2020 | No relevant outcome data | Lin, 2021 | No relevant outcome data | Sheshadri 2019 | No relevant outcome data |
| Correa, 2020 | No relevant outcome data | Liu, 2023 | No relevant outcome data | Soares, 2017 | No relevant outcome data |
| Daniilidis 2004 | No relevant outcome data | Lopes, 2019 | No relevant outcome data | Soliman, 2015 | No relevant outcome data |
| Dashtidehkordi, 2019 | No relevant outcome data | Luo, 2023 | No relevant outcome data | Suh, 2002 | Not RCT |
| de Lima, 2013 | No relevant outcome data | Manfredini, 2009 | No relevant outcome data | Suhardjono 2019 | No relevant outcome data |
| De Paul, 2002 | No relevant outcome data | Manfredini, 2015 | No relevant outcome data | Suzuki, 2017 | Did not meet criteria |
| Deligiannis, 1999a | No relevant outcome data | Manfredini, 2017 | No relevant outcome data | Tabibi, 2023 | No relevant outcome data |
| Deligiannis, 1999b | No relevant outcome data | Marchesan, 2014 | No relevant outcome data | Tapia González, 2021 | No relevant outcome data |
| Deus, 2021 | No relevant outcome data | Marinho, 2016 | No relevant outcome data | Tentori, 2010 | Not RCT |
| Dipp, 2018 | No relevant outcome data | Martin-Alemany, 2016 | No relevant outcome data | Tsai, 2015 | Did not meet criteria |
| Dipp, 2020 | No relevant outcome data | Martin-Alemany, 2020 | No relevant outcome data | Tsuyuki, 2003 | No relevant outcome data |
| de Olival Fernandes, 2018 | No relevant outcome data | Martinez-Olmos, 2021 | No relevant outcome data | Turoń-Skrzypińsk, 2023 | No relevant outcome data |
| do Valle, 2019 | No relevant outcome data | Marzougui, 2022 | No relevant outcome data | van den Ham 2007 | No relevant outcome data |
| Dong, 2011 | No relevant outcome data | Matsumoto, 2007 | No relevant outcome data | Van Vilsteren, 2004 | No relevant outcome data |
| Dong, 2019 | No relevant outcome data | McGregor, 2018 | No relevant outcome data | Vrakas, 2017 | No relevant outcome data |
| Dziubek, 2016 | No control group | Medeiros, 2019 | No relevant outcome data | Young, 2020 | Duplicate of Graham-Brown |
| Esteve Simo, 2015 | No relevant outcome data | Meléndez-Oliva, 2022 | No control group | Watanabe, 2021 | No relevant outcome data |
| Exel, 2021 | No relevant outcome data | Mihaescu, 2013 | Not RCT | Wilund, 2010 | No relevant outcome data |
| Feldkötter, 2021 | Did not meet criteria | Moeinzadeh 2022 | No relevant outcome data | Wu, 2014 | No relevant outcome data |
| Ferreira, 1999 | No relevant outcome data | Mohseni, 2013 | No relevant outcome data | Yabe, 2021 | No relevant outcome data |
| Figueiredo, 2012 | No relevant outcome data | Momeni, 2014 | No relevant outcome data | Yamaguchi, 2021 | Not RCT |
| Fitts, 1999 | No relevant outcome data | Mortazavi, 2013 | No relevant outcome data | Yeh, 2020 | No relevant outcome data |
| Frey, 1999 | No relevant outcome data | Moug, 2004 | Not RCT | YurtKuran, 2007 | No relevant outcome data |
| Fuzari, 2019 | No control group | Moura, 2020 | No relevant outcome data | Zhao, 2017 | No control group |
| Gadelha, 2021 | No relevant outcome data | Myers, 2021 | No relevant outcome data | Zhao, 2020 | No relevant outcome data |

**Supplemental Table 4:** Detailed Exercise Intervention Characteristics

| **Study** | **Modality** | **Duration**  **(weeks)** | **Frequency**  **(sessions/**  **week)** | **Total Time**  **per session** | **Intensity / information** |
| --- | --- | --- | --- | --- | --- |
| Chen J, 2010 [1] | Resistance, seated, via ankle cuffs. Supervised | 24 | 2  Intradialytic | NS (included 5 min WU & 5 min CD) | Moderate, 60% 1RM RPE (13/20) with progression, (2 sets, 8 repetitions, 8 exercises) |
| Dobsak, 2012 [2] Aerobic Training | Aerobic, cycle ergometer. Supervised | 20 | 3  Intradialytic (second half of dialysis session) | 20 min ↑ after 5 weeks to 40 min (includes 5 min WU & 5 min CD) | Moderate, 60% of peak ergonomic tolerance level |
| Frih, 2017 [3] | Combined training. Aerobic, various apparati. Resistance, low weight, multigym. Supervised. | 16 | 4  Interdialytic | 60 min (includes 10 min WU & 10 min CD) | AT: Moderate, 20 mins with progression and RPE  RT: 50%1RM (12-15 repetitions) ↑ by 5% monthly, Moderate intensity with progression and RPE |
| Giannaki, 2013 [4] | Aerobic, cycle ergometer. Supervised | 26 | 3  Intradialytic | 45-60 mins | Moderate, 60-65% of MEC with progression |
| Graham-Brown, 2021 [5] | Aerobic, cycle ergometer. Supervised | 26 | 3  Intradialytic | 30 mins | Moderate, RPE (12-14/20) with progression |
| Greenwood, 2021 [6] | Combined training. Aerobic, cycle ergometer  Resistance, upper and lower body exercises, ankle weights, TheraBand. Supervised | 26 | AT: 3  RT: 2 Intradialytic (first half of dialysis session) | AT: 21min ↑ 30 min ↑ 40 min  RT on completion of AT twice weekly | AT: Light with progression  RT: Light, lower limb exercises (3 sets, 10-15 repetitions) with progression |
| Hristea, 2016 [7] | Aerobic, cycle ergometer. Supervised | 26 | 3  Intradialytic (first half of dialysis session) | ↑ 30 min | Light, RPE (3/10) with progression |
| Huang, 2020 [8] | Combined training. Aerobic, cycle ergometer. Resistance via increase in cycling difficulty. Supervised | 24 | 3  Intradialytic (first half of dialysis session) | 30 mins including 10-20 min AT combined with 10-20 mins RT with different ratios at different stages of trial. (Plus 5 min WU & 5 min CD – 40 mins total). | Moderate, RPE (12-14/20) with progression |
| Jamshidpour, 2020 [9] | Combined training. Aerobic, cycle ergometer. Resistance via increase in cycling difficulty. Supervised | 8 | 3  Intradialytic (first half of dialysis session) | 3-5 min WU. AT: 20 min ↑ 45 min. RT: varied | AT: Vigorous, RPE (11-15/20) with progression  RT: Vigorous, 60% of 3 RM (8-12 repetitions), RPE (15/20 as a maximum) with progression |
| Jeong, 2019 [10] | Aerobic, cycle ergometer. Supervised | 52 | 3 Intradialytic (first half of dialysis session) | 30 min ↑ 45 min (includes 5-10 min WU) | Moderate, RPE (12-14/20) with progression |
| Kim, 2022 [11] | Aerobic, cycle ergometer. Supervised | 12 | 3  Intradialytic (first half of dialysis session) | 40 - 70 min (includes 5 min WU & 5 min CD) | Vigorous, RPE (12-15/20) with progression |
| Koh, 2010 [12]  Centre based | Aerobic, cycle ergometer. Supervised | 26 | 3  Intradialytic  (first half of dialysis session) | 15min ↑ 30 min ↑ 45 min | Moderate, RPE (12-13/20) with progression |
| Koh, 2010 [12]  Home based | Aerobic, walking. Unsupervised | 26 | 3  Interdialytic | 15 min ↑ 30 min ↑ 45 min | Moderate, RPE (12-13/20) with progression |
| Lee, 2020 [13]  Aerobic training | Aerobic, cycle ergometer. Supervised | 12 | 3  Intradialytic | 40 mins (includes 5 min WU and 5 mins CD) | Moderate, RPE (11-13/20) with progression |
| Lee, 2020 [13]  Resistance training | Resistance, TheraBand and soft weights. Supervised | 12 | 3  Interdialytic | 30-40 mins (includes 5 min WU and 5 mins CD) | Moderate, RPE (13-15/20) with progression. 9 exercises |
| Lee, 2020 [13]  Combined training | Combined training. Aerobic, cycle ergometer. Resistance, TheraBand and soft weights. Supervised | 12 | 3  Intradialytic | 70-70 mins (includes AT of 40 mins (includes 5 min WU and 5 mins CD) and 30-40 mins RT after the AT | AT: Moderate, RPE (11-13/20) with progression  RT: Moderate, RPE (13-15/20) with progression. 9 exercises |
| Matsufuji, 2015 [14] | Aerobic, sit to stand. Supervised | 12 | 3  Interdialytic  (immediately prior to dialysis session) | 15 mins | Moderate, 5 sets of 50% of max duration at baseline, no progression |
| Maynard, 2019 [15] | Combined training. Aerobic: ergometer and virtual reality games. Resistance: ankle weights and TheraBand. Supervised | 12 | 3  Intradialytic (first half of dialysis session) | 30-60 mins | Moderate, RPE (12-14/20) with progression every 2 weeks |
| Molsted, 2004 [16] | Combined training. Aerobic: cycle ergometer, step training. Resistance: circuit training. Supervised | 20 | 2  Interdialytic | 60 mins. 10 min WU, 20-30 mins various aerobic and resistance exercises, 15-20 min cycling, 5-10 mins CD | Vigorous, RPE (14-17/20) with progression and intensity adjustment weekly |
| Ouzouni, 2009 [17] | Combined training. Aerobic: cycle ergometer. Resistance: ankle weights and TheraBand. Supervised | 40 | 3  Intradialytic (first half of dialysis session) | 60-90 mins (includes 5 min WU and 5 mins CD). AT:30 mins, RT N/S | Moderate, RPE (13-14/20) with progression for AT and ↑ sets and repetitions for RT |
| Pereira, 2022 [18] | Aerobic, cycle ergometer. Supervised | 12 | 3  Intradialytic (first half of dialysis session) | 30 mins (includes 5 min WU and 5 mins CD) | Moderate, RPE (3-4/10) with progression |
| Rosa, 2018 [19] | Resistance, free weights and TheraBand. Supervised | 12 | 3  Intradialytic  (1 session immediately prior to dialysis, 1 during) | 40-50 mins completed over 2 sessions | Vigorous, progressive ↑ intensity by ↑ weight (2 sets, 15-20 repetitions, 11 exercises) until fatigued |
| Samara, 2016 [20] | Aerobic, swimming. Supervised | 16 | 3  Interdialytic | 60 mins (includes 10 min WU & 10 min CD) | Moderate, RPE (13-14/20) with progression |
| Song, 2012 [21] | Resistance, TheraBand, sandbags. Supervised | 12 | 3  Interdialytic  (prior to dialysis session) | 30 mins (includes 5 min WU and 5 mins CD) | Moderate, RPE (11-15/20) with progression (3 sets, 10 - 15 repetitions, 12 exercises) |
| Thompson, 2016 [22] | Aerobic, cycle ergometer. Supervised | 12 | 3  Intradialytic (second half of dialysis session) | 15 min ↑ 2.5 minutes each week to 45 min (includes 5 min WU & 5 min CD) | Moderate, RPE (12-14/20) with progression |
| Thompson, 2016 [22] | Resistance, ankle weights and TheraBand. Supervised | 12 | 3  Intradialytic (second half of dialysis session) | 45-60 min (included 5 min WU & 5 min CD) | Moderate, RPE (12-14/20) with progression (3 sets, 10-15 repetitions, 4 exercises) |
| Thompson, 2016 [22] | Combined training. Aerobic: cycle ergometer. Resistance: ankle weights and TheraBand. Supervised | 12 | 3  Intradialytic (second half of dialysis session) | 45-75 min (included aerobic cycling of 15 min ↑ 2.5 minutes each week to 45 min, 15-30 min resistance training and 5 min WU & 5 min CD) | Moderate, RPE (12-14/20) with progression |
| Uchiyama, 2019 [23] | Combined training, home based. Aerobic, walking. Resistance, TheraBand. Unsupervised | 12 | 3  Interdialytic | AT: 20min ↑ 30 min | AT: Moderate, RPE (11-13/20) with progression walking thrice weekly, 40-60% HRM  RT: Moderate, 70% 1 RM (1 set, 10 repetitions) twice weekly with progression |
| Yuenyongchaiwat, 2021 [24] | Inspiratory muscle training, Power Breathe K5 device. Supervised | 8 | 3  Intradialytic | 4.30 – 6 minutes | Moderate with progression 40% MIP (3 sets, 15 maximal inspirations) with ↑ in load and inspirations after 28 days |
| Zhang, 2020 [25] | Resistance, wrist and ankle weights. Supervised | 12 | 2-3  Intradialytic  (first half of dialysis session) | 40-50 mins (includes 5 min WU and 5 mins CD). | Moderate, RPE (10-13/20) with progression Weeks 1-4: 2/week (2 sets, 8-10 repetitions)  Weeks 5-12: 3/week (3 sets, 11-12 repetitions) |

**AT**=aerobic training; **CD**=cool-down; **CT**=combined training; **HRM**=heart rate maximum; **Hz**=Hertz; **max**=maximum; **mA**=milliamps; **MEC**=maximal exercise capacity based on maximal O_2_ consumption and corresponding heart rate; **min**=minute; **MIP**=Maximal Inspiratory Pressure; **RM**=repetition maximum; **RPE**=Borg Rating of Perceived Exertion scale; **RT**=resistance training; ***u*s**=millisecond; **WU**=warm-up; **↑**=increase; **↓**=decrease

**Supplemental Table 5:** Brief summary of sub-analyses indicating if exercise led to a significant improvement in outcomes in favour of the intervention group

| ***Sub-analyses by:*** | PCS | MCS |
| --- | --- | --- |
| ***Exercise modality*** | | |
| - **Aerobic Training** | ✓ | ✓ |
| - **Resistance Training** | ✓ | ✓ |
| - **Combined Training** | ✓ | 🗶 |
| - **Inspiratory Muscle Training** | 🗶 | 🗶 |
| ***Intensity of exercise*** | | |
| - **Light** | 🗶 | ✓ |
| - **Moderate** | ✓ | ✓ |
| - **Vigorous** | ✓ | 🗶 |
| ***Duration of exercise intervention*** | | |
| - **>4 to 12 weeks** | ✓ | 🗶 |
| - **>12 to 26 weeks** | ✓ | ✓ |
| - **>26 weeks** | 🗶 | 🗶 |
| ***Schedule of exercise intervention*** | | |
| - **Interdialytic** | ✓ | ✓ |
| - **Intradialytic** | ✓ | ✓ |
| ***Supervision of exercise intervention*** | | |
| - **Supervised** | ✓ | ✓ |
| - **Unsupervised** | 🗶 | 🗶 |
| ***Patient-reported outcome measure*** | | |
| - **Health-Related Quality of Life Short Form Questionnaire** | ✓ | ✓ |
| - **Kidney Disease Quality of Life Questionnaire** | ✓ | 🗶 |

**Supplemental Table 6:** Cochrane risk of bias tool for randomised controlled trials (Rob2)

| **Study ID** | **Randomisation process** | **Deviations from the intended** | **Missing outcome** | **Measurement of the outcome** | **Selection of the reported result** | **Overall** |
| --- | --- | --- | --- | --- | --- | --- |
| Chen J, 2010 [1] |  |  |  |  |  |  |
| Dobsak, 2012 [2] |  |  |  |  |  |  |
| Frih (2017) [3] |  |  |  |  |  |  |
| Giannaki, 2013 [4] |  |  |  |  |  |  |
| Graham-Brown, 2021 [5] |  |  |  |  |  |  |
| Greenwood, 2021 [6] |  |  |  |  |  |  |
| Hristea, 2016 [7] |  |  |  |  |  |  |
| Huang, 2020 [8] |  |  |  |  |  |  |
| Jamshidpour, 2020 [9] |  |  |  |  |  |  |
| Jeong (2019) [10] |  |  |  |  |  |  |
| Kim, 2022 [11] |  |  |  |  |  |  |
| Koh, 2010 [12] |  |  |  |  |  |  |
| Lee, 2020 [13] |  |  |  |  |  |  |
| Matsufuji, 2015 [14] |  |  |  |  |  |  |
| Maynard, 2019 [15] |  |  |  |  |  |  |
| Molsted, 2004 [16] |  |  |  |  |  |  |
| Ouzouni, 2009 [17] |  |  |  |  |  |  |
| Pereira, 2022 [18] |  |  |  |  |  |  |
| Rosa, 2018 [19] |  |  |  |  |  |  |
| Samara, 2016 [20] |  |  |  |  |  |  |
| Song, 2012 [21] |  |  |  |  |  |  |
| Thompson, 2016 [22] |  |  |  |  |  |  |
| Uchiyama, 2019 [23] |  |  |  |  |  |  |
| Yuenyongchaiwat, 2021 [24] |  |  |  |  |  |  |
| Zhang, 2020 [25] |  |  |  |  |  |  |

|  | = Low risk |  |  |
| --- | --- | --- | --- |

**Supplemental Table 7:** Assessment of study quality and reporting using Tool for the assessment of study quality and reporting in exercise (TESTEX)

| Study | Eligibility criteria specified | Randomisation  details specified | Allocation concealed | Groups similar at baseline | Assessors blinded | Outcomes measures assessed >85% participants# | Intention to treat analysis | Reporting between group statistical comparison* | Point measures & measures of variability | Activity monitoring in control group | Relative exercise intensity constant | Exercise volume & energy expenditure | Overall TESTEX (/15) |
| --- | --- | --- | --- | --- | --- | --- | --- | --- | --- | --- | --- | --- | --- |
| Chen J, 2010 [1] | 1 | 0 | 1 | 1 | 0 | 2 | 1 | 2 | 1 | 0 | 1 | 1 | 11 |
| Dobsak, 2012 [2] | 1 | 0 | 0 | 1 | 0 | 1 | 0 | 2 | 1 | 1 | 1 | 1 | 9 |
| Frih (2017) [3] | 1 | 1 | 1 | 1 | 1 | 1 | 0 | 2 | 1 | 0 | 1 | 1 | 11 |
| Giannaki, 2013 [4] | 1 | 1 | 1 | 1 | 1 | 2 | 0 | 0 | 1 | 0 | 1 | 1 | 10 |
| Graham-Brown, 2021 [5] | 1 | 1 | 1 | 1 | 1 | 3 | 1 | 2 | 1 | 0 | 1 | 1 | 14 |
| Greenwood, 2021 [6] | 1 | 1 | 1 | 1 | 1 | 2 | 1 | 2 | 1 | 0 | 1 | 1 | 13 |
| Hristea, 2016 [7] | 1 | 0 | 1 | 1 | 0 | 1 | 0 | 2 | 1 | 0 | 1 | 1 | 9 |
| Huang, 2020 [8] | 1 | 1 | 1 | 1 | 1 | 1 | 1 | 2 | 1 | 0 | 1 | 1 | 12 |
| Jamshidpour, 2020 [9] | 1 | 1 | 1 | 1 | 1 | 2 | 0 | 2 | 1 | 0 | 1 | 1 | 12 |
| Jeong (2019) [10] | 1 | 1 | 1 | 1 | 0 | 3 | 0 | 2 | 1 | 0 | 1 | 1 | 12 |
| Kim, 2022 [11] | 1 | 1 | 1 | 1 | 0 | 3 | 1 | 2 | 1 | 0 | 1 | 1 | 13 |
| Koh, 2010 [12] | 1 | 1 | 1 | 1 | 0 | 2 | 0 | 2 | 1 | 0 | 1 | 1 | 11 |
| Lee, 2020 [13] | 1 | 0 | 1 | 1 | 0 | 1 | 0 | 2 | 1 | 0 | 1 | 1 | 9 |
| Matsufuji, 2015 [14] | 1 | 1 | 1 | 1 | 1 | 2 | 0 | 2 | 1 | 0 | 1 | 1 | 12 |
| Maynard, 2019 [15] | 1 | 1 | 1 | 1 | 0 | 2 | 0 | 2 | 1 | 0 | 1 | 1 | 11 |
| Molsted, 2004 [16] | 1 | 1 | 1 | 1 | 1 | 1 | 0 | 2 | 1 | 0 | 1 | 1 | 11 |
| Ouzouni, 2009 [17] | 1 | 0 | 1 | 1 | 0 | 2 | 0 | 2 | 1 | 0 | 1 | 1 | 10 |
| Pereira, 2022 [18] | 1 | 1 | 1 | 1 | 1 | 0 | 0 | 2 | 1 | 0 | 1 | 1 | 10 |
| Rosa, 2018 [19] | 1 | 1 | 1 | 1 | 0 | 3 | 1 | 2 | 1 | 0 | 1 | 1 | 13 |
| Samara, 2016 [20] | 1 | 1 | 1 | 1 | 0 | 3 | 0 | 2 | 1 | 0 | 1 | 1 | 12 |
| Song, 2012 [21] | 1 | 1 | 1 | 1 | 0 | 3 | 0 | 2 | 1 | 0 | 1 | 1 | 12 |
| Thompson, 2016 [22] | 1 | 1 | 1 | 1 | 1 | 2 | 1 | 2 | 1 | 1 | 1 | 1 | 14 |
| Uchiyama, 2019 [23] | 1 | 1 | 1 | 1 | 0 | 3 | 1 | 2 | 1 | 0 | 1 | 1 | 13 |
| Yuenyongchaiwat, 2021 [24] | 1 | 0 | 1 | 1 | 1 | 2 | 0 | 2 | 1 | 1 | 1 | 1 | 12 |
| Zhang, 2020 [25] | 1 | 1 | 1 | 1 | 0 | 2 | 0 | 2 | 1 | 0 | 1 | 1 | 11 |
| Total |  |  |  |  |  |  |  |  |  |  |  | Median score = 11 | |

Key: total out of 15 points. Legend: #three points possible—one point if adherence >85%, one point if adverse events reported, one point if exercise attendance is reported. *Two points possible—one point if primary outcome is reported, one point if all other outcomes reported. 0 awarded if no mention was made of this criterion, or if it was unclear.

**References**

1. Chen JLT, Godfrey S, Ng TT, Moorthi R, Liangos O, Ruthazer R, Jaber BL, Levey AS, Castaneda-Sceppa C (2010) Effect of intra-dialytic, low-intensity strength training on functional capacity in adult haemodialysis patients: a randomized pilot trial. Nephrology, dialysis, transplantation 25:1936-1943. <https://doi.org/10.1093/ndt/gfp739>

2. Dobsak P, Homolka P, Svojanovsky J, Reichertova A, Soucek M, Novakova M, Dusek L, Vasku J, Eicher J-C, Siegelova J (2012) Intra-Dialytic Electrostimulation of Leg Extensors May Improve Exercise Tolerance and Quality of Life in Hemodialyzed Patients. Artificial organs 36:71-78. <https://doi.org/10.1111/j.1525-1594.2011.01302.x>

3. Frih B, Jaafar H, Mkacher W, Salah ZB, Hammami M, Frih A (2017) The effect of interdialytic combined resistance and aerobic exercise training on health related outcomes in chronic hemodialysis patients: The Tunisian randomized controlled study. Frontiers in physiology 8:288-288. <https://doi.org/10.3389/fphys.2017.00288>

4. Giannaki CD, Sakkas GK, Karatzaferi C, Hadjigeorgiou GM, Lavdas E, Kyriakides T, Koutedakis Y, Stefanidis I (2013) Effect of exercise training and dopamine agonists in patients with uremic restless legs syndrome: a six-month randomized, partially double-blind, placebo-controlled comparative study. BMC nephrology 14:194-194. <https://doi.org/10.1186/1471-2369-14-194>

5. Graham-Brown MPM, March DS, Young R, Highton PJ, Young HML, Churchward DR, Dungey M, Stensel DJ, Bishop NC, Brunskill NJ, Smith AC, McCann GP, McConnachie A, Burton JO (2021) A randomized controlled trial to investigate the effects of intra-dialytic cycling on left ventricular mass. Kidney International 99:1478-1486. <https://doi.org/https://doi.org/10.1016/j.kint.2021.02.027>

6. Greenwood SA, Koufaki P, Macdonald JH, Bhandari S, Burton JO, Dasgupta I, Farrington K, Ford I, Kalra PA, Kean S, Kumwenda M, Macdougall IC, Messow C-M, Mitra S, Reid C, Smith AC, Taal MW, Thomson PC, Wheeler DC, White C, Yaqoob M, Mercer TH (2021) Randomized Trial—PrEscription of intraDialytic exercise to improve quAlity of Life in Patients Receiving Hemodialysis. Kidney international reports 6:2159-2170. <https://doi.org/10.1016/j.ekir.2021.05.034>

7. Hristea D, Deschamps T, Paris A, Lefrançois G, Collet V, Savoiu C, Ozenne S, Coupel S, Testa A, Magnard J (2016) Combining intra-dialytic exercise and nutritional supplementation in malnourished older haemodialysis patients: Towards better quality of life and autonomy. Nephrology (Carlton, Vic) 21:785-790. <https://doi.org/10.1111/nep.12752>

8. Huang M, Lv A, Wang J, Zhang B, Xu N, Zhai Z, Gao J, Wang Y, Li T, Ni C (2020) The effect of intradialytic combined exercise on hemodialysis efficiency in end-stage renal disease patients: a randomized-controlled trial. International urology and nephrology 52:969-976. <https://doi.org/10.1007/s11255-020-02459-1>

9. Jamshidpour B, Bahrpeyma F, Khatami M-R (2020) The effect of aerobic and resistance exercise training on the health related quality of life, physical function, and muscle strength among hemodialysis patients with Type 2 diabetes. Journal of bodywork and movement therapies 24:98-103. <https://doi.org/10.1016/j.jbmt.2019.10.003>

10. Jeong JH, Biruete A, Tomayko EJ, Wu PT, Fitschen P, Chung HR, Ali M, McAuley E, Fernhall B, Phillips SA, Wilund KR (2019) Results from the randomized controlled IHOPE trial suggest no effects of oral protein supplementation and exercise training on physical function in hemodialysis patients. Kidney international 96:777-786. <https://doi.org/10.1016/j.kint.2019.03.018>

11. Kim S, Park H-J, Yang D-H (2022) An intradialytic aerobic exercise program ameliorates frailty and improves dialysis adequacy and quality of life among hemodialysis patients: a randomized controlled trial. Kidney research and clinical practice 41:462-472. <https://doi.org/10.23876/j.krcp.21.284>

12. Koh KP, Fassett RG, Sharman JE, Coombes JS, Williams AD (2010) Effect of Intradialytic Versus Home-Based Aerobic Exercise Training on Physical Function and Vascular Parameters in Hemodialysis Patients: A Randomized Pilot Study. American journal of kidney diseases 55:88-99. <https://doi.org/10.1053/j.ajkd.2009.09.025>

13. Lee J-Y, Baek, S-H, Lee, Y-M, Cho J-H, Kim, J-C, Choi, S-W (2020) The effect of a 12-week intradialytic exercise on patients' blood indicies, physical performance, and quality of life. Journal of Medical Imaging and Health Informatics 10:1429-1435. <https://doi.org/10.1166/jmihi.2020.3066>

14. Matsufuji SM, Shoji TMDP, Yano Y, Tsujimoto YMDP, Kishimoto HMD, Tabata TMD, Emoto MMDP, Inaba MMDP (2015) Effect of Chair Stand Exercise on Activity of Daily Living: A Randomized Controlled Trial in Hemodialysis Patients. Journal of renal nutrition 25:17-24. <https://doi.org/10.1053/j.jrn.2014.06.010>

15. Maynard LG, de Menezes DL, Lião NS, de Jesus EM, Andrade NLS, Santos JCD, da Silva Júnior WM, Bastos KA, Barreto Filho JAS (2019) Effects of Exercise Training Combined with Virtual Reality in Functionality and Health-Related Quality of Life of Patients on Hemodialysis. Games Health J 8:339-348. <https://doi.org/10.1089/g4h.2018.0066>

16. Molsted S, Eidemak I, Helle Tauby S, Jens Halkjaer K (2004) Five Months of Physical Exercise in Hemodialysis Patients: Effects on Aerobic Capacity, Physical Function and Self-Rated Health. Nephron 96:c76. <https://doi.org/10.1159/000076744>

17. Ouzouni S, Kouidi E, Sioulis A, Grekas D, Deligiannis A (2009) Effects of intradialytic exercise training on health-related quality of life indices in haemodialysis patients. Clinical rehabilitation 23:53-63. <https://doi.org/10.1177/0269215508096760>

18. Pereira ABN, Santana LL, Rocha LDB, Cunha KDC, Rocha LSDO, Santos MCDS, Normando VMF, Torres DDC, Rocha RSB (2022) Physical Exercise Affects Quality of Life and Cardiac Autonomic Modulation in Patients With Chronic Kidney Failure Submitted to Hemodialysis: A Randomized Clinical Trial. Perceptual and motor skills 129:696-713. <https://doi.org/10.1177/00315125221085811>

19. Rosa C, Nishimoto DY, Souza GDe, Ramirez AP, Carletti CO, Daibem CGL, Sakkas GK, Monteiro HL (2018) Effect of continuous progressive resistance training during hemodialysis on body composition, physical function and quality of life in end-stage renal disease patients: a randomized controlled trial. Clinical rehabilitation 32:899-908. <https://doi.org/10.1177/0269215518760696>

20. Samara A, Kouidi E, Fountoulakis K, Alexiou S, Deligiannis A (2016) The effects of aquatic exercise on functional capacity and health-related quality of life in hemodialysis patients. Journal of Clinical and Experimental ephrology 1:15. <https://doi.org/10.21767/2472-5056.100015>

21. Song WJ, Sohng KY (2012) Effects of progressive resistance training on body composition, physical fitness and quality of life of patients on hemodialysis. J Korean Acad Nurs 42:947-956. <https://doi.org/10.4040/jkan.2012.42.7.947>

22. Thompson S, Klarenbach S, Molzahn A, Lloyd A, Gabrys I, Haykowsky M, Tonelli M (2016) Randomised factorial mixed method pilot study of aerobic and resistance exercise in haemodialysis patients: DIALY-SIZE. BMJ open 6:e012085-e012085. <https://doi.org/10.1136/bmjopen-2016-012085>

23. Uchiyama K, Washida N, Morimoto K, Muraoka K, Kasai T, Yamaki K, Miyashita K, Wakino S, Itoh H (2019) Home-based Aerobic Exercise and Resistance Training in Peritoneal Dialysis Patients: A Randomized Controlled Trial. Scientific reports 9:2632-2632. <https://doi.org/10.1038/s41598-019-39074-9>

24. Yuenyongchaiwat K, Namdang P, Vasinsarunkul P, Phongsukree P, Chaturattanachaiyaporn K, Pairojkittrakul S, Traitanon O (2021) Effectiveness of inspiratory muscle training on respiratory fitness and breathlessness in chronic renal failure: A randomized control trial. Physiotherapy research international : the journal for researchers and clinicians in physical therapy 26:e1879-n/a. <https://doi.org/10.1002/pri.1879>

25. Zhang F, Huang L, Wang W, Shen Q, Zhang H (2020) Effect of intradialytic progressive resistance exercise on physical fitness and quality of life in maintenance haemodialysis patients. Nursing open 7:1945-1953. <https://doi.org/10.1002/nop2.585>
